# A structural and functional bioinformatics study of QTY-designed retinylidene proteins

**DOI:** 10.1017/qrd.2025.10009

**Published:** 2025-07-14

**Authors:** Siqi Pan

**Affiliations:** Independent Researcher

**Keywords:** bioinformatics, computational studies on functional properties of biological molecule, GPCRs, membrane protein structure, protein dynamics simulation

## Abstract

Retinylidene proteins are retinal-binding light-sensitive proteins found in organisms ranging from microbes to human. Microbial opsins have been utilized in optogenetics, while animal opsins are essential for vision and light-dependent metabolic functions. However, retinylidene proteins have hydrophobic transmembrane (TM) domains, which makes them challenging to study. In this structural and functional bioinformatics study, I use the QTY (glutamine, threonine, tyrosine) code to design water-soluble QTY analogues of retinylidene proteins, including nine human and three microbial opsins. I provide superpositions of the AlphaFold3-predicted hydrophobic native proteins and their water-soluble QTY analogues, and experimentally determined structures when available. I also provide a comparison of surface hydrophobicity of the variants. Despite significant changes to the protein sequence (35.53–50.24% in the TM domain), protein characteristics and structures are well preserved. Furthermore, I run molecular dynamics (MD) simulations of native and QTY-designed OPN2 (rhodopsin) and analyze their response to the isomerization of 11-*cis*-retinal to all-*trans*-retinal. The results show that the QTY analogue has similar functional behavior to the native protein. The findings of this study indicate that the QTY code can be used as a robust tool to design water-soluble retinylidene proteins. These have potential applications in protein studies, therapeutic treatments, and bioengineering.

## Introduction

Retinylidene proteins are photochemically reactive proteins that are bound to or can bind to retinal (vitamin A aldehyde) as their chromophore (Spudich *et al.*, 2000). For convenience, I will use the term ‘opsin’ interchangeably with ‘retinylidene protein’, despite the fact that ‘opsin’ sometimes refers specifically to the chromophore-free apoprotein. Retinylidene proteins are divided into two groups, animal opsins and microbial opsins, which are evolutionarily distinct but share common characteristics such as having seven transmembrane (TM) domains and a retinal-binding lysine residue (Spudich *et al.*, 2000; Yee *et al.*, 2013). In this study, I will consider nine animal opsins and three microbial opsins.

*Animal opsins* are a type of class A GPCR (G-protein-coupled receptor), which are characterized by seven transmembrane domains, the NPxxY motif, and activation of G proteins through the outward movement of TM6 (Zhou *et al.*, 2019). Almost all animal opsins include a lysine residue that forms a Schiff base link with the retinal (Gühmann *et al.*, 2022). When retinal absorbs a photon, it isomerizes, usually changing from 11-*cis* to all-*trans.* The subsequent conformational changes of the protein are well studied using bovine rhodopsin, which changes from dark state to BATHO state, then LUMI, META I, and META II (Okada *et al.*, 2001). Proton transfer plays an important role in this process (Mahalingam *et al.*, 2008). Afterward, the protein bleaches and returns to the dark state, completing what is called the photocycle. As the protein conformation changes to META, TM6 moves characteristically outward, activating the G protein. In photoreceptor cells, the G protein, transducin, activates a phosphodiesterase, which hydrolyzes cGMP into GMP, decreasing the activity of cGMP-gated cation channels and hyperpolarizing the cell (Chabre and Deterre, 1989).

In this study, I select nine opsins expressed in the human nervous system: OPN1MW (UniProt ID: P04001), OPN1LW (UniProt ID: P04001), OPN1SW (UniProt ID: P03999), OPN2 (UniProt ID: P04001), OPN3 (UniProt ID: Q9H1Y3), OPN4 (UniProt ID: Q9UHM6), OPN5 (UniProt ID: Q6U736), RGR (UniProt ID: P47804), and RRH (UniProt ID: O14718). They belong to several evolutionarily distinct families of animal opsins (Terakita, 2005).

*OPN1MW* (medium-wave-sensitive opsin 1), *OPN1LW* (long-wave-sensitive opsin 1), and *OPN1SW* (short-wave-sensitive opsin 1) are expressed in retinal cone photoreceptors and are responsible for color vision (Bowmaker and Dartnall, 1980). Certain variants of OPN1MW, OPN1LW, and OPN1SW, respectively, cause deuteranopia, protanopia, and tritanopia, which are different types of color blindness (Baraas *et al.*, 2012; Ueyama *et al.*, 2002). The absence of both functional OPN1MW and OPN1LW causes blue cone monochromacy, an X-linked congenital cone dysfunction syndrome (Wissinger *et al.*, 2022), and cone dystrophy 5, an X-linked cone dystrophy (Gardner *et al.*, 2010).

*OPN2* (opsin 2), also known as rhodopsin, is expressed in retinal rod photoreceptors and is responsible for vision at low light intensity (Hubbard and Kropf, 1958). Certain variants lead to autosomal recessive or autosomal dominant retinitis pigmentosa and congenital stationary night blindness (Fanelli *et al.*, 2021). OPN2 is a representative animal opsin, one of the first studied. In fact, bovine OPN2 is the first opsin to be sequenced (Nathans and Hogness, 1984), as well as the first GPCR whose crystal structure was resolved experimentally (Palczeski *et al.*, 2000). Many studies on the functional mechanisms of animal opsins also focus on OPN2. Consequently, I chose OPN2 to conduct a functional analysis in this study in order to further explore the effectiveness of the QTY code in redesigning retinylidene proteins.

*OPN3* (opsin 3), also known as encephalopsin or panopsin, is activated by blue and ultraviolet A light. It was discovered in the brain (Blackshaw and Snyder, 1999). It is also expressed in melanocytes and keratinocytes in the skin and regulates functions such as melanogenesis, cell differentiation, and glucose uptake. Its expression is also found in the liver, pancreas, kidney, lung, heart, and skeletal muscles. When expressed in neurons, the release of neurotransmitters is inhibited by light, making OPN3 a useful inhibitory optogenetic tool (Copits *et al.*, 2021).

*OPN4* (opsin 4), also known as melanopsin, is expressed in ipRGC (intrinsically photosensitive retinal ganglion cells) in the ganglion cell layer in the retina (Provencio *et al.*, 1998). It is essential for non-image-forming responses to light, including the pupillary reflex, optokinetic visual tracking response, and photoentrainment and regulation of circadian rhythm. Certain variants of OPN4 lead to seasonal affective disorder and other circadian rhythm disorders (Berson *et al.*, 2002). Rendering OPN4-containing ipRGCs capable of image formation is also a potential pathway for the treatment of various eye diseases such as retinitis pigmentosa and diabetic retinopathy.

*OPN5* (opsin 5), also known as neuropsin, is activated by blue and ultraviolet A light (Tarttelin *et al.*, 2003). It is expressed in the retina and contributes to the regulation of light-dependent vascular development and photoentrainment in the cornea and retina (Buhr *et al.*, 2015). Certain variants of OPN5 may lead to cycloplegia, paralysis of the ciliary muscle in the eye.

*RGR* (RPE-retinal GPCR) is expressed in RPE (retinal pigmented epithelium) and Müller cells in the retina (Shen *et al.*, 1994). Unlike the aforementioned human opsins, RGR preferentially binds all-*trans*-retinal and may catalyze its isomerization into 11-*cis*-retinal via a retinochrome-like mechanism. It is expressed only in tissue surrounding photoreceptors and plays a role in the light-dependent synthesis of visual chromophore (Radu *et al.*, 2008).

*RRH* (RPE-derived rhodopsin homolog), also known as peropsin, is localized in the microvilli of RPE cells that surround photoreceptor outer segments (Sun *et al.*, 1997). It is another protein that preferentially binds to all-*trans*-retinal (Cook *et al.*, 2017). Although not much information is known about RRH, it can reasonably be inferred that it photoisomerizes all-*trans*-retinal to 11-*cis*-retinal and may play a role in the upkeep of photoreceptor functions.

*Microbial opsins* are transmembrane ion pumps or channels (Findlay and Pappin, 1986). They are also 7TM, though this is due to convergent evolution rather than homology (Yee *et al.*, 2013). The chromophore, retinal, usually isomerizes from all-*trans* to 13-*cis*, different from the case of animal opsins (Findlay and Pappin, 1986). In addition, microbial opsins often form oligomers to carry out their functions (Gmelin *et al.*, 2007).

In this study, I select three microbial opsins: BACR (UniProt ID: P02945), BACH (UniProt ID: B0R2U4), and ChR2 (UniProt ID: Q8RUT8).

*BACR* (bacteriorhodopsin) is a light-driven proton pump. It is one of the first microbial opsins discovered (Oesterhelt and Stoeckenius, 1971). *BACH* (halorhodopsin) is a light-driven chloride pump activated by yellow light (Schobert and Lanyi, 1982). *ChR2* (channelrhodopsin 2) is a light-activated sodium channel activated by blue light (Nagel *et al.*, 2003). BACH and ChR2 are among the first optogenetic tools (Han and Boyden, 2007; Zhang *et al.*, 2007). BACH is used for inhibition, while ChR2 is used for excitation.

I have asked whether retinylidene proteins could be redesigned to be more soluble. Retinylidene proteins are all integral membrane proteins with seven transmembrane alpha helices embedded in a lipid bilayer. Because of the hydrophobic properties of the transmembrane domains, they are not water-soluble without the aid of detergents.

Bacteriorhodopsin has been the subject of several protein-solubilizing studies, though with limited success (Qing *et al.*, 2022). Recently, researchers leveraged a neural network, SolubleMPNN, which was built upon ProteinMPNN, to engineer soluble variants of bacteriorhodopsin while maintaining its ligand-binding ability and light-sensing function (Nikolaev *et al.*, 2025).

Instead of taking a computational approach, I apply the QTY (glutamine, threonine, tyrosine) code to systematically engineer water-soluble analogues with reduced hydrophobicity in membrane proteins. There are structural similarities between hydrophobic and polar amino acids: leucine (L) and glutamine (Q); isoleucine (I)/valine (V) and threonine (T); and phenylalanine (F) and tyrosine (Y), as can be observed on high-resolution electron density maps (Tegler *et al.*, 2020; Zhang and Egli, 2022; Zhang *et al.*, 2018). This justifies the replacement of hydrophobic amino acids with polar ones. Zhang *et al.* initially applied the QTY code to design several detergent-free chemokine receptors, all of which retained structural thermal stability and native ligand-binding and enzymatic activities despite substantial changes to the transmembrane domain (Tegler *et al.*, 2020; Zhang *et al.*, 2018). Zhang’s team also applied the QTY code to design water-soluble GPCRs, including chemokine receptors (Qing *et al.*, 2019; Skuhersky *et al.*, 2021; Tegler *et al.*, 2020; Zhang *et al.*, 2018), cytokine receptors (Hao *et al.*, 2020), and olfactory receptors (Johnsson *et al.*, 2025; Skuhersky *et al.*, 2021). In addition to GPCR, they used the QTY code to design water-soluble glucose transporters (Smorodina *et al.*, 2022), ABC transporters (Pan *et al.*, 2024), monoamine neurotransmitter transporters (Karagöl *et al.*, 2024c), glutamate transporters (Karagöl *et al.*, 2024a, 2024b), mitochondrial megacomplex (Chen and Zhang, 2025), antibodies (Li *et al.*, 2024b), potassium channels (Smorodina *et al.*, 2024), receptor kinases (Li *et al.*, 2024a), transmembrane enzymes (Chen *et al.*, 2025), as well as bacterial enzymes with beta barrel structures (Sajeev-Sheeja and Zhang, 2024; Sajeev-Sheeja *et al.*, 2023). The QTY-designed water-soluble CXCR4 chemokine receptor was successfully used in biomimetic sensors (Qing *et al.*, 2023).

AlphaFold2 was released by Google DeepMind in July 2021 (Jumper *et al.*, 2021). It has greatly facilitated the study of QTY-designed protein variants. Subsequently, AlphaFold3 was released in May 2024, featuring improved architecture and improved efficiency (Abramson *et al.*, 2024). Furthermore, AlphaFold3 enabled the accurate prediction of complexes of multiple proteins, as well as complexes with modified residues, nucleic acids, ions, and certain ligands. QTY studies have made use of these new features (Chen and Zhang, 2025; Johnsson *et al.*, 2025).

GROMACS is a molecular dynamics (MD) simulation program released in 1995 (Berendsen *et al.*, 1995). Its 5.0 version was released in 2015 (Abraham *et al.*, 2015). GROMACS enables an efficient and realistic simulation of biomolecular systems and has been used to investigate the structural and functional properties of QTY-designed protein variants in various studies (Johnsson *et al.*, 2025; Karagöl *et al.*, 2024b; Li *et al.*, 2024a, 2024b; Smorodina *et al.*, 2024).

In this article, I apply the QTY code to redesign nine human opsins and three microbial opsins. I provide the superpositions of the AlphaFold3-predicted hydrophobic native proteins and their water-soluble QTY variants, and experimentally determined structures when available. I also provide a comparison of the surface hydrophobicity of the variants. Furthermore, I run MD simulations of native and QTY-designed OPN2 and analyze their response to the 11-*cis* to all-*trans* isomerization of the retinal chromophore.

## Methods

### QTY design, protein sequence alignment, and other characteristics

The native protein sequences for OPN1MW, OPN1LW, OPN1SW, OPN2, OPN3, OPN4, OPN5, RGR, RRH, BACR, BACH, and ChR2 were obtained from UniProt (https://www.uniprot.org). The sequence for ChR2 was truncated according to the experimentally determined structure in the RCSB PDB (PDB ID: 8ZAN). The QTY designs were performed through the protein solubilizing server (https://pss.sjtu.edu.cn/) (Tao *et al.*, 2022). For ChR2, the secondary structure was provided to the server in SS3 format according to the RCSB PDB structure.

### AlphaFold3 predictions

I predicted the structures of native proteins and their QTY variants using the AlphaFold3 website (https://alphafoldserver.com) (Abramson *et al.*, 2024). For microbial opsins, the dimers and trimers have identical subunits, that is, are homodimers and homotrimers. In AlphaFold3, this was achieved by altering the number of protein copies.

### Structure superpositions

PDB files for native protein structures determined experimentally by X-ray diffraction or electron microscopy were taken from RCSB PDB: OPN2 (PDB ID: 5W0P), BACR (PDB ID: 7XJC), BACH (PDB ID: 2JAF), ChR2 (PDB ID: 8ZAN). The AlphaFold3-predicted native and QTY variants were taken directly from the most probable predicted structure. Superpositions were performed via the command ‘super’ in PyMOL (https://pymol.org). I removed unstructured loops at the N and C terminals for the sake of clarity.

### Structure visualization

I used PyMOL (https://pymol.org) to superpose the native predicted protein structures, their QTY variants, and the experimentally determined structures for the proteins where these existed. I then used UCSF ChimeraX (https://www.cgl.ucsf.edu/chimerax/) to render each protein model with hydrophobicity patches.

### Molecular dynamics simulations

All MD simulations and analyses were executed on a desktop computer with Intel Xeon Platinum 8352 V Processor, 256 GB RAM, and 2 NVIDIA GPUs (GeForce RTX 4090) with 24 GB VRAM each. All MD simulations were conducted using GROMACS 2024.5 (Abraham *et al.*, 2015) with the CHARMM36m all-atom force field (Huang *et al.*, 2017). Data for retinal were obtained from NAMD Wiki (https://www.ks.uiuc.edu/Research/namd/wiki/index.cgi?RetinalTop for topology and https://www.ks.uiuc.edu/Research/namd/wiki/index.cgi?RetinalPar for parameters) and manually integrated into the protein file. Initial structures, configuration files, and command-line codes for the simulations are publicly available on Zenodo (https://doi.org/10.5281/zenodo.15377505).

Regarding native OPN2, the protein membrane system was constructed using the web-based membrane builder CHARMM-GUI and downloaded in GROMACS format (Jo *et al.*, 2008; Lee *et al.*, 2016; Wu *et al.*, 2014). The protein was centered in a rectangular box. The generated membrane models consisted of 40% POPC, 40% POPE, 10% POPS, and 10% cholesterol, which simulated a rod photoreceptor disk membrane where native OPN2 is usually expressed (Albert and Boesze-Battaglia, 2005). The system was solvated in TIP3P water with 150 mM NaCl. NaCl was used instead of KCl to simulate the environment of rod photoreceptor outer segments (Govardovskii, 1971). 11-*cis*-Retinal was manually added to the protein files. LINCS constraints were used for constraints, and the Verlet integrator was used. Electrostatics was handled with particle mesh Ewald (PME), with both Coulomb and van der Waals interaction cutoffs set at 1.2 nm. The energy of the system was minimized using the steepest descent until the maximum forces converged below 1000 kJ/mol/nm. The standard six-step CHARMM-GUI NP 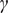 T equilibration protocol (Jo *et al.*, 2008) was used, with two 125-ps NVT equilibration simulations, and one 125-ps and three 500-ps NP 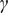 T equilibration simulations. Temperature and pressure were maintained at 303.15 K and 1.0 bar, respectively, using the V-rescale thermostat and C-rescale barostat with surface tension coupling. Following equilibration, a 10-ns production MD simulation was run. The retinal molecule was then manually switched to the all-*trans* state by rotating C13 to C15 and associated hydrogen and methyl groups by 180 degrees around the C11–C12 bond. This mimicked the effect of the absorption of a photon by 11-*cis*-retinal. Energy minimization and equilibration were repeated exactly as before, and then a 120-ns production MD simulation was run.

Regarding QTY-designed OPN2, I found that helix 9 of the QTY analogue flung up and adhered to the surface that was originally the transmembrane domain, which disrupted the normal conformation. Therefore, this helix was truncated. The system was constructed directly using GROMACS. The protein was centered in a rectangular box with dimensions equal to those of native OPN2. The equilibration involved a sequence of simulations: one 250-ps NVT equilibration, followed by one 125-ps and one 1500-ps NPT equilibration, totaling an equilibration duration that matches that of the natural OPN2. All other configurations and parameters were identical to those of the original OPN2.

After the simulations, I extracted frames from the trajectory to inspect the structures before and after retinal isomerization. I also calculated the RMSD (‘gmx rms’ command), number of hydrogen bonds (‘gmx hbond’ command) and water molecules (‘gmx select’ command) inside the binding pocket, interaction energy (including short-range Coulombic interaction energy and short-range Lennard-Jones energy) (‘gmx energy’ command), the RMSF (‘gmx rmsf’ command), and radius of gyration (‘gmx gyrate’ command). The detailed commands are publicly available in the online database for this study.

## Results and discussion

### The rationale of the QTY code

The transmembrane segments of membrane proteins are hydrophobic in nature. This leads to a challenge to study their structure and function. Traditionally, detergent must be used to purify them. Zhang *et al.* considered a different approach, that is, via systematic soluble protein design. There exist structural similarities, as can be observed on high-resolution electron density maps, between certain hydrophobic and hydrophilic amino acids: leucine (L) and glutamine (Q), isoleucine (I) or valine (V) and threonine (T), and phenylalanine (F) with tyrosine (Y). This fact enables systematic replacement of L with Q, I/V with T, and F with Y in all transmembrane segments of the membrane protein, which Zhang *et al.* named the QTY code. QTY analogues are less hydrophobic than native membrane proteins. Although their amino acid sequences are significantly changed, they still exhibit relatively preserved structure, isoelectric points (pI), and molecular weights (MW) compared to the native membrane proteins (Table 1).

**Table 1.** Protein characteristics of 12 retinylidene proteins and their QTY analogs

Name	RMSD (Å)	pl	MW (kDa)	TM variation (%)	Overall variation (%)
OPN1MW	–	8.90	40.58	–	–
OPN1MW^QTY^	0.468	8.82	40.85	41.86	19.78
OPN1LW	–	8.89	40.57	–	–
OPN1LW^QTY^	0.611	8.83	40.76	40.12	18.96
OPN1SW	–	8.75	38.72	–	–
OPN1SW^QTY^	0.486	8.66	39.30	46.51	23.19
OPN2	–	6.20	38.89	–	–
OPN2^QTY^	0.559	6.20	39.29	46.58	21.55
OPN3	–	9.29	44.87	–	–
OPN3^QTY^	0.454	9.18	45.47	49.12	20.90
OPN4	–	9.35	52.64	–	–
OPN4^QTY^	0.307	9.19	53.08	50.34	15.48
OPN5	–	9.11	39.73	–	–
OPN5^QTY^	0.555	9.02	40.04	45.58	18.93
RGR	–	8.34	31.87	–	–
RGR^QTY^	0.537	8.29	32.39	42.86	21.65
RRH	–	8.77	37.42	–	–
RRH^QTY^	0.537	8.29	32.39	42.86	21.65
BACR	–	4.75	26.92	–	–
BACR^QTY^	0.448	4.75	27.40	46.67	25.30
BACH	–	5.34	26.96	–	–
BACH^QTY^	0.296	5.34	27.50	44.19	30.04
ChR2	–	6.13	34.90	–	–
ChR2^QTY^	0.235	6.13	35.30	35.53	22.22

MW, molecular weight; pI, isoelectric focusing; RMSD, root-mean-square distance; TM, transmembrane.

### Protein sequence alignments and other characteristics of retinylidene proteins

**Figure 1.**
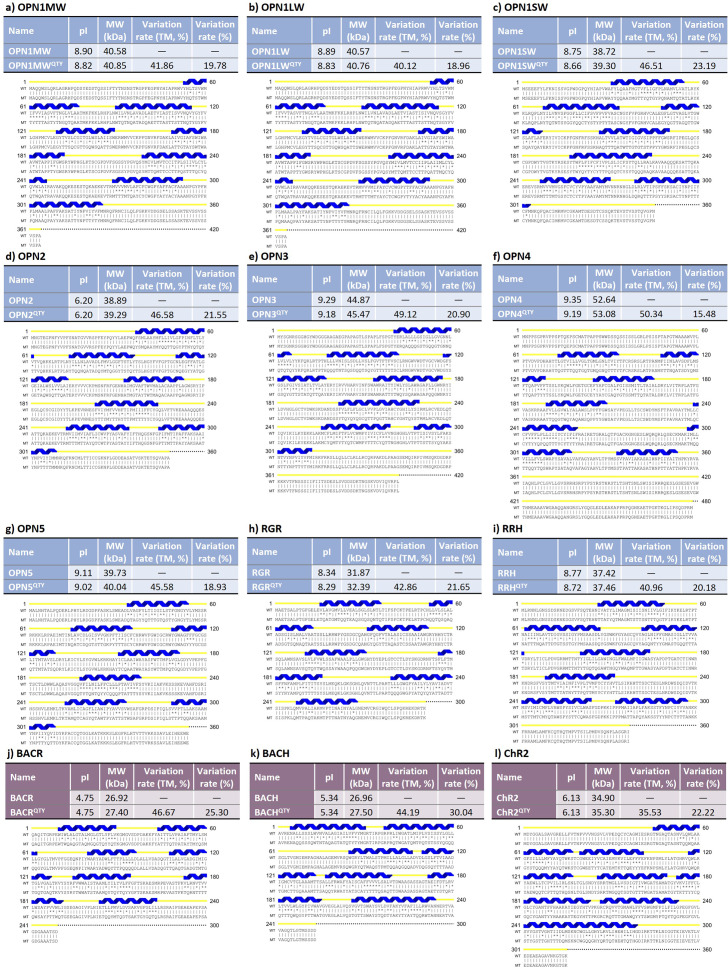
The protein sequence alignments of 12 retinylidene proteins and their QTY analogs. Blue tables: human opsins; purple tables: microbial opsins. The symbols  and 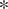 indicate that amino acids are identical or different, respectively. Amino acids L, I/V, and F in TM (transmembrane) alpha helices (shown in blue above the sequences) are replaced with Q, T, and Y, respectively. The variation in the TM domain ranges from 35.53% to 50.24%, while the overall variation rate ranges from 15.48% to 30.04%. The characteristics of the proteins are shown above the sequences. Despite large variation rates, the pI only experiences a slight change between 0.00 and 0.16 and the MW increases slightly by a value between 0.04 and 0.60 kDa. The enlarged individual sequence alignments are available in supplementary information. The alignments are (*a*) OPN1MW versus OPN1MW 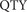 , (*b*) OPN1LW versus OPN1LW 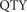 , (*c*) OPN1SW versus OPN1SW 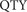 , (*d*) OPN2 versus OPN2 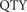 , (*e*) OPN3 versus OPN3 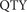 , (*f*) OPN4 versus OPN4 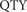 , (*g*) OPN5 versus OPN5 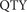 , (*h*) RGR versus RGR 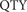 , (*i*) RRH versus RRH 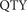 , (*j*) BACR versus BACR 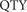 , (*k*) BACH versus BACH 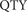 , and (*l*) ChR2 versus ChR2 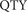 .

The protein sequences of nine human opsins and three microbial opsins were aligned with their QTY analogues (Figure 1). The QTY substitution resulted in an overall variation of the sequence of 15.48% to 30.04% and a variation of the transmembrane domain of 35.53% to 50.24%. Despite changes in amino acid composition and sequence, the pI only experienced a slight change, between 0.00 and 0.16. This is because the amino acids Q, T, and Y have neutral charges and do not introduce additional charges to the system. The MW increased slightly by a value between 0.04 and 0.60 kDa. This is because the hydrophilic amino acids usually have nitrogen or oxygen atoms in place of carbon atoms in hydrophobic acids and thus may have higher mass: L (131.17 Da) versus Q (146.14 Da); I (131.17 Da) and V (117.15 Da) versus T (119.12 Da); F (165.19 Da) versus Y (181.19 Da).

### Superpositions of AlphaFold3-predicted native human opsins, their water-soluble QTY variants, and experimentally determined structures

**Figure 2.**
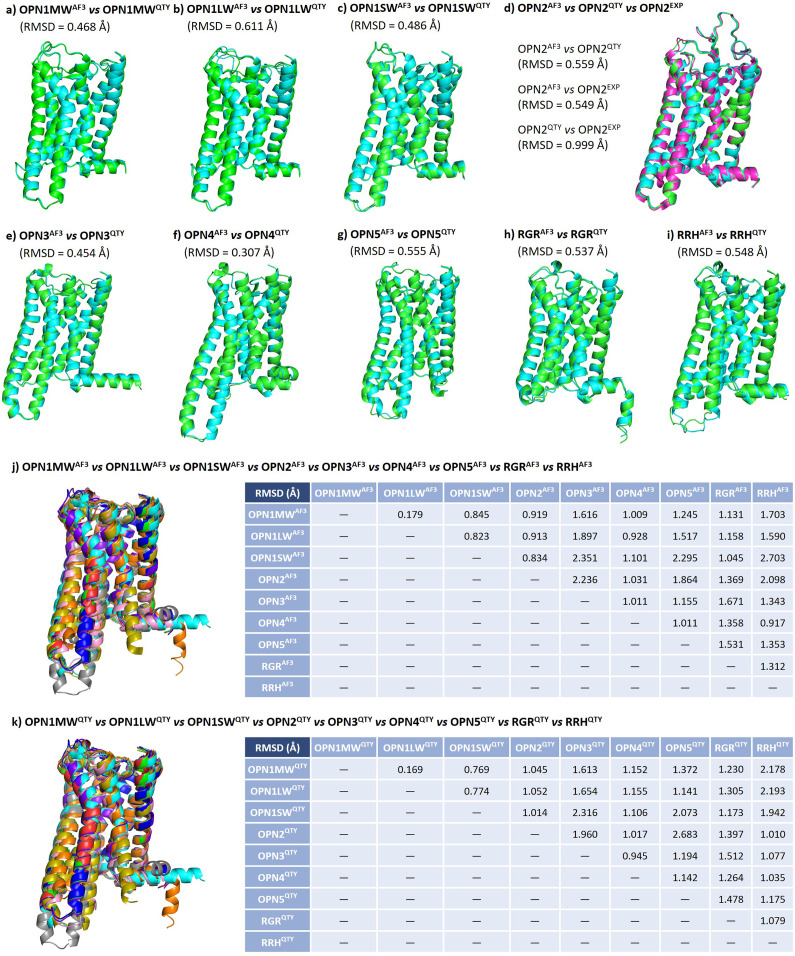
Superposition of AlphaFold3-predicted native human retinylidene proteins, their QTY analogs, and experimentally determined structures. For clarity, unstructured N- and C-terminal ends are deleted. For (a) to (i), despite significant changes in the protein sequence, the structures superpose very well. The root-mean-square distance (RMSD) values are quite small, from 0.307 to 0.611 Å, with only one exception (OPN2 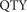 vs. OPN2 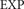 , RMSD = 0.999 Å). Green: AlphaFold3-predicted native structure; cyan: AlphaFold3-predicted QTY analog structure; magenta: experimentally determined structure. The superpositions are (*a*) OPN1MW 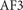 versus OPN1MW 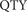 , (*b*) OPN1LW 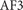 versus OPN1LW 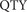 , (*c*) OPN1SW 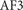 versus OPN1SW 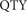 , (*d*) OPN2 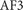 versus OPN2 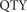 versus OPN2 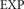 , (*e*) OPN3 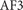 versus OPN3 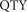 , (*f*) OPN4 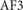 versus OPN4 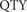 , (*g*) OPN5 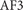 versus OPN5 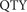 , (*h*) RGR 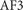 versus RGR 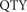 , and (*i*) RRH 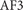 versus RRH 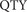 . For (*j*) and (*k*), there is a large degree of similarity between the RMSD between a pair of native proteins and that between the corresponding pair of QTY analogs. Green: OPN1MW; red: OPN1LW; blue: OPN1SW; purple: OPN2; cyan: OPN3; gray: OPN4; olive: OPN5; orange: RGR; pink: RRH. The superpositions are (*j*) OPN1MW 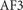 versus OPN1LW 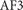 versus OPN1SW 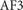 versus OPN2 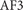 versus OPN3 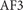 versus OPN4 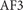 versus OPN5 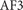 versus RGR 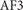 versus RRH 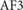 and (*k*) OPN1MW 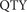 versus OPN1LW 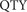 versus OPN1SW 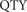 versus OPN2 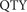 versus OPN3 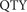 versus OPN4 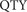 versus OPN5 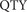 versus RGR 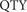 versus RRH 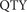 .

Although the chromophore, retinal, is bound to retinylidene proteins in an internal pocket, it is only incorporated into the protein after protein folding is complete. In other words, protein folding does not depend on the chromophore. Thus, it is justified to predict the ligand-free forms of the proteins. I used AlphaFold3 to predict the structure of the nine native human opsins and their water-soluble QTY analogues. The structures superposed very well (Figure 2*a*–*i*). For OPN2, which has an available X-ray diffraction structure, I also superposed the experimentally determined structure with the AlphaFold3-predicted structures. Overall, the root mean square distances (RMSD) were small, from 0.307 to 0.611 Å, with only one exception of OPN2 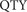 versus OPN2 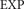 (RMSD = 0.999 Å). This shows that the water-soluble QTY analogues are quite similar to native proteins in terms of structure.

All nine of these human opsins belong to the same large family, that is, animal opsins. However, they belong to different subfamilies and have structural differences from each other. In order to investigate the extent to which the water-soluble QTY analogues preserve these differences, I also conducted pairwise superpositions within all nine native human opsins and within all nine QTY analogues of human opsins (Figure 2*j* and *k*). There was a large degree of similarity between the RMSD between a pair of native proteins and that between the corresponding pair of QTY analogues. Therefore, I conclude that the structural differences are relatively well preserved before and after the QTY substitution. However, whether the functional differences are preserved remains a problem to study.

### Superpositions of AlphaFold3-predicted native microbial opsins, their water-soluble QTY variants, and experimentally determined structures

**Figure 3.**
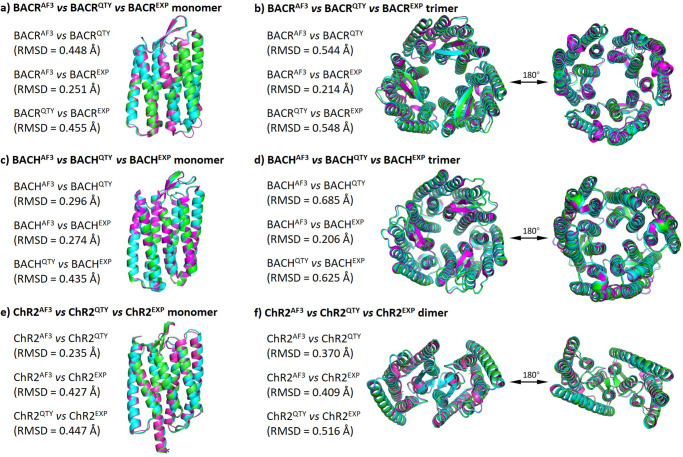
Superposition of AlphaFold3-predicted native microbial retinylidene proteins, their QTY analogs, and experimentally determined structures. Despite significant changes in the protein sequence, the structures superpose very well. The root-mean-square distance (RMSD) values are quite small, with the highest being 0.685 Å. For clarity, unstructured N- and C-terminal ends are deleted. Green: AlphaFold3-predicted native structure; cyan: AlphaFold3-predicted QTY analog structure; magenta: experimentally determined structure. The superpositions are (*a*) BACR 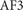 versus BACR 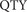 versus BACR 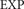 monomer, (*b*) BACR 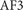 versus BACR 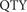 versus BACR 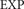 trimer, (*c*) BACH 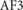 versus BACH 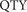 versus BACH 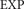 monomer, (*d*) BACH 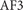 versus BACH 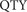 versus BACH 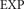 trimer, (*e*) ChR2 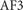 versus ChR2 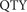 versus ChR2 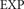 monomer, and (*f*) ChR2 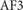 versus ChR2 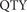 versus ChR2 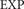 dimer.

I used AlphaFold3 to predict the structure of monomers of the three native microbial opsins, their water-soluble QTY analogues. Since microbial opsins often have to form oligomers to be functional, I also predicted the structure of native and QTY variants of BACR trimers, BACH trimers, and ChR2 dimers. These predicted structures were also superposed with experimentally determined structures (X-ray diffraction or electron microscopy). The structures superposed very well (Figure 3). Overall, the root-mean-square distances (RMSD) were small, with the highest value being 0.685 Å. This shows that the water-soluble QTY analogues are likely capable of forming oligomers with structures similar to those of native proteins.

### Analysis of the hydrophobic surface of native retinylidene proteins and their water-soluble QTY variants

As transmembrane proteins, retinylidene proteins require detergents to be separated from the lipid bilayer. The detergents act as an interface between the hydrophobic transmembrane domain of the protein and the surrounding water, thus solubilizing the protein. If these proteins are exposed in water without detergents, their hydrophobicity will induce them to aggregate and precipitate, with their structures and functions disrupted.

**Figure 4.**
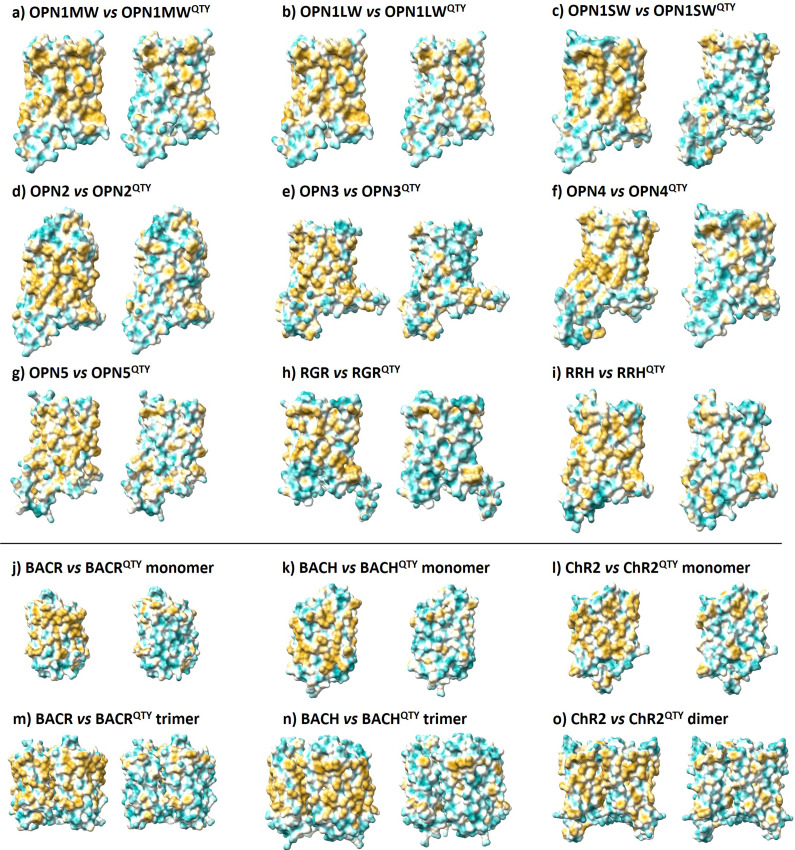
Hydrophobic surface of 12 retinylidene proteins and their water-soluble QTY analogs. Hydrophobic patches are shown in yellow, while hydrophilic patches are shown in cyan. The native proteins have many hydrophobic patches due to the presence of hydrophobic amino acids, including L, I, V, and F. After QTY substitution, hydrophilic Q, T, and Y have respectively replaced hydrophobic L, I/V, and F, and the hydrophobic patches in the surface of transmembrane helices have become more hydrophilic. In addition, the surface shape of the native and QTY analogs are very similar. For clarity, unstructured N- and C-terminal ends are deleted. The comparisons are (*a*) OPN1MW versus OPN1MW 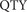 , (*b*) OPN1LW versus OPN1LW 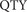 , (*c*) OPN1SW versus OPN1SW 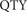 , (*d*) OPN2 versus OPN2 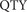 , (*e*) OPN3 versus OPN3 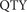 , (*f*) OPN4 versus OPN4 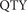 , (*g*) OPN5 versus OPN5 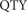 , (*h*) RGR versus RGR 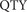 , (*i*) RRH versus RRH 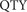 , (*j*) BACR versus BACR 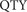 monomer, (*k*) BACH versus BACH 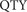 monomer, (*l*) ChR2 versus ChR2 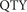 monomer, (*m*) BACR versus BACR 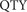 trimer, (*n*) BACH versus BACH 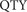 trimer, and (*o*) ChR2 versus ChR2 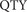 dimer.

The hydrophobic portions of the protein surface are represented in yellow (Figure 4). In the native proteins, there are large hydrophobic patches in the transmembrane domains, which are embedded within the lipid bilayer. The nonpolar and hydrophobic amino acids, including leucine (L), isoleucine (I), valine (V), phenylalanine (F), alanine (A), methionine (M), and tryptophan (W), interact with the hydrophobic hydrocarbon chains and expel water.

I investigated the surface hydrophobicity of water-soluble QTY analogues in comparison with the native proteins. After applying the QTY code to, respectively, replace the hydrophobic amino acids L, I/V, and F with hydrophilic amino acids glutamine (Q), threonine (T), and tyrosine (Y), the hydrophobic surface areas were significantly reduced. More importantly, since the electron density maps of the corresponding amino acids are similar, the alpha helices in QTY analogues had a similar contour to those in the native proteins. The QTY analogues successfully retained their structural integrity and stability.

### AlphaFold3 predictions

DeepMind released AlphaFold3 in May 2024, marking a significant leap in accuracy for modeling across biomolecular space. This latest iteration outperforms state-of-the-art docking tools and its predecessor, AlphaFold-Multimer v.2.3, in protein structure and protein–protein interaction predictions (Abramson *et al.*, 2024). AlphaFold3 reduces the reliance on multiple sequence alignment by integrating a diffusion-based model, enabling it to predict a broader spectrum of biomolecules, including ligands, ions, nucleic acids, modified residues, and large protein megacomplexes. On October 9, 2024, DeepMind’s founders, Demis Hassabis and John Jumper, received the Nobel Prize in Chemistry for revolutionizing protein structure prediction through leveraging machine learning.

AlphaFold3 is easily accessible online (https://alphafoldserver.com), allowing users to make 30 predictions a day at present. The structures of the QTY analogues were predicted using the AlphaFold3 server, which was free of charge, and the results were produced within a few minutes.

DeepMind also collaborated with the EBI to make over 214 million predicted protein structures available through the AlphaFold Protein Structure Database (https://alphafold.ebi.ac.uk). This number is continuously expanding, with the quality of predictions further improving with the advent of AlphaFold 3.

However, AlphaFold3 still has limitations that have been encountered in this study. Although AlphaFold3 allows the structural prediction of complexes that include small-molecule ligands, it only supports a small range of ligands, which does not include retinal. I was therefore unable to predict the structure of chromophore-bound states of retinylidene proteins with AlphaFold3. Consequently, I chose another approach, namely molecular dynamics simulation, in order to investigate the behavior of QTY opsin analogues when they are bound to the chromophore.

### *Simulation of 11-*cis *to all-*trans *isomerization of retinal in native OPN2 and its QTY analogue*

QTY substitutions do not introduce changes to the essential NPxxY motif or the retinal-binding lysine of human retinylidene proteins. Therefore, I proposed that QTY analogues of human opsins may have conserved functions and carried out a molecular dynamics (MD) simulation. I recorded and analyzed the behavior of native OPN2 and its QTY analogue before and after the 11-*cis* to all-*trans* isomerization. Note that my aim is not to simulate the whole photocycle of OPN2, which takes longer than 1 ms (Fanelli *et al.*, 2021), but rather to find proof that the QTY analogue conserves the function of being activated by isomerization of retinal.

**Figure 5.**
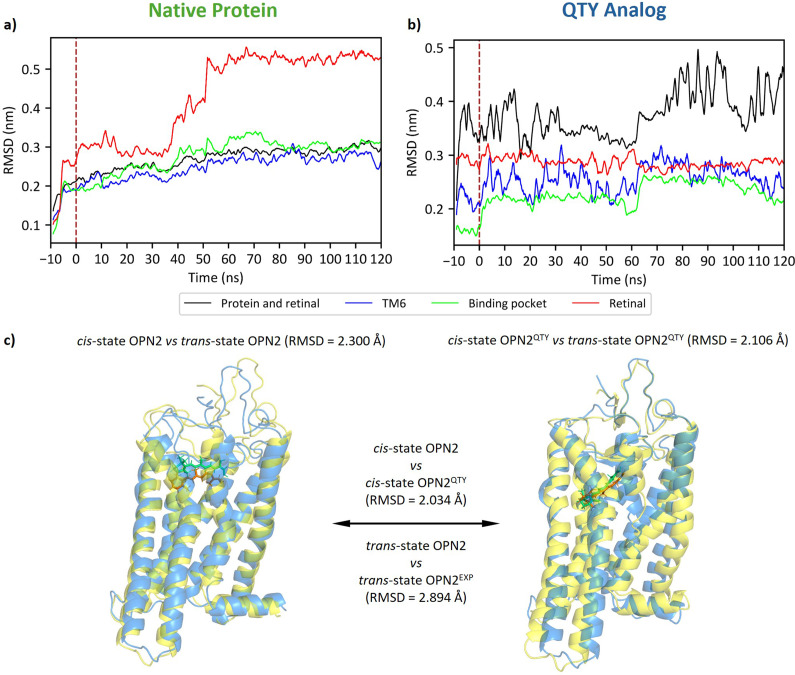
The conformational changes of native OPN2 and its QTY analog before and after 11-*cis* to all-*trans* isomerization of the chromophore, retinal. (*a*, *b*) 1 ns running averages of the root-mean-square distances (RMSD) of the protein–retinal complex, transmembrane helix 6 (TM6), the retinal-binding pocket, and retinal. By convention, the isomerization is set at time 0 ns, which is indicated by a brown, vertical dashed line. (*c*) Superpositions between *cis*-state OPN2, *trans*-state OPN2, *cis*-state OPN2 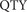 , and *trans*-state OPN2 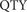 . Both OPN2 and OPN2 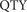 exhibit conformational changes, with RMSDs greater than 2 Å. Blue: *cis*-state protein, orange: 11-*cis*-retinal; yellow: *trans*-state protein; greenish cyan: all-*trans* retinal.

The MD simulation provided evidence of conformational change in both native and QTY analogues (Figure 5). The native protein experienced a slower change from its original conformation, while the QTY analogue showed more fluctuations and sudden changes. Under further inspection, this phenomenon might be due to the entrance of water molecules into the QTY analogue. In addition, the retinal molecule had a greater degree of change in conformation in the native protein than in the QTY analogue. Nevertheless, the retinal in the QTY analogue was still able to cause changes to surrounding residues, sometimes doing so via intervening water molecules. It may be noted that the RMSD was still fluctuating, which means that the protein had not yet stabilized. This was expected, since the OPN2 was still in the process of transition from BATH to LUMI state, and this provided evidence for the activation of the protein in response to isomerization of retinal.

**Figure 6.**
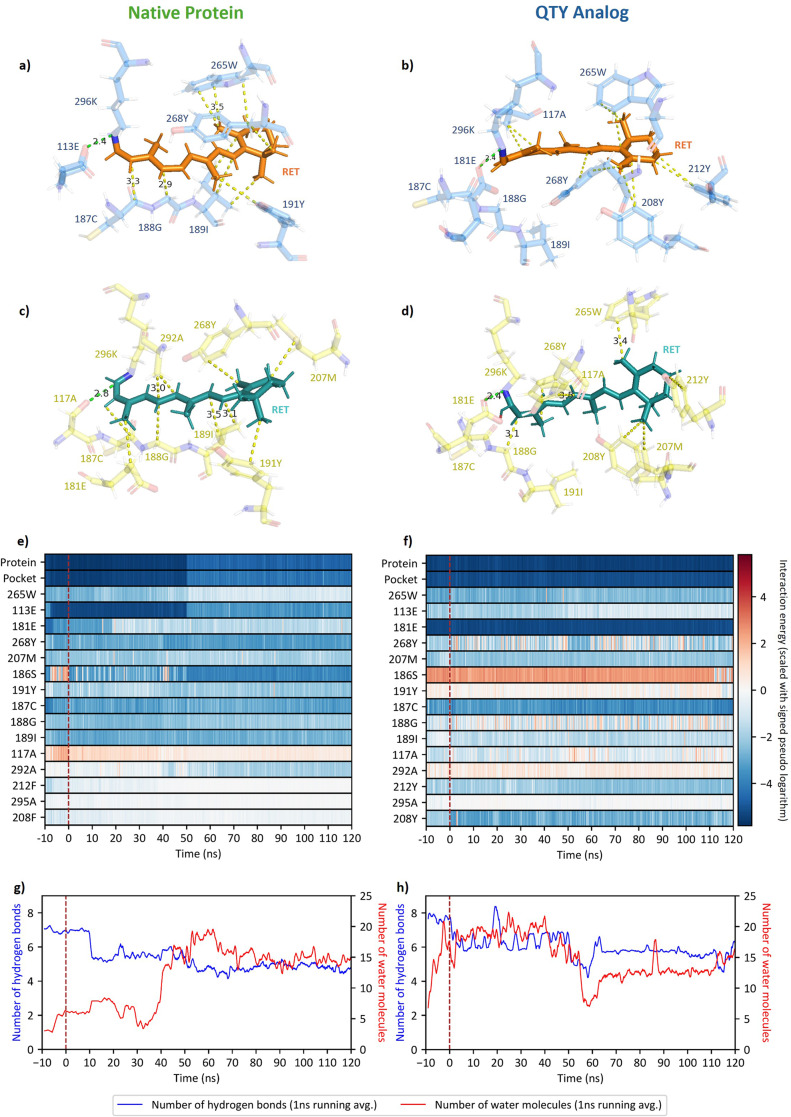
Changes in the retinal-binding pocket and protein–ligand interaction in native OPN2 and its QTY analog before and after 11-*cis* to all-*trans* isomerization of the chromophore. (*a*, *b*) Close-ups of the binding pocket in *cis*-state. Protein–ligand interactions with lengths shorter or equal to 3.5 Å are shown in the figure. Blue: protein residues, orange: 11-*cis*-retinal; green dashed lines: ion bridge; yellow dashed lines: van der Waals and/or hydrophobic interactions. (*c*, *d*) Close-ups of the binding pocket in *trans*-state. Protein–ligand interactions with lengths shorter or equal to 3.5 Å are shown in the figure. Yellow: protein residues, greenish cyan: all-*trans*-retinal; green dashed lines: ion bridge; yellow dashed lines: van der Waals and/or hydrophobic interactions. (*e*, *f*) The interaction energies (IEs) between the protein, the binding pocket, individual residues, and retinal. IE is the sum of the short-range Coulombic interaction energy and short-range Lennard–Jones energy. Note that IE is a product of MD simulation and is not necessarily a ‘real’ physical quantity. For clarity, IE is rescaled using the signed pseudo logarithm ( 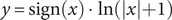 ). By convention, the isomerization is set at time 0 ns, which is indicated by a brown, vertical dashed line. The large changes in IE in OPN2 around 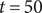 ns are due to the entrance of water molecules into the binding pocket, near 113E. The QTY analog has more negative IE at residues 208 and 212 due to F to Y substitutions. Besides from these, the similarity between the IE of OPN2 and OPN2 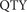 is a strong suggestion of functional conservation. (*g*, *h*) The number of hydrogen bonds formed between residues in the binding pocket, and the number of water molecules within the binding pocket. By convention, the isomerization is set at time 0 ns, which is indicated by a brown, vertical dashed line.

Zooming in on the retinal-binding pocket, I observed that retinal is held within its binding pocket by several forces in both the native and QTY analogue: the counterions of the retinal Schiff base (RSB) (113E and 181E), the hydrophobic interactions with neighboring residues (e.g., 265W, 268Y, and the 187–189 beta strand), and, sometimes, steric collisions (Figure 6). Since retinal is a ligand that binds in an internal pocket, I was unable to calculate the Gibbs free energy of its binding. Nevertheless, the distributions of interaction energies were found to be similar in native OPN2 and the water-soluble QTY analogue, except for small differences: native OPN2 experienced a change in interaction energy at about 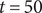 ns due to the entrance of water near 113E; the QTY analogue had more negative interaction energy at residues 208 and 212 due to phenylalanine (F) to tyrosine (Y) substitutions. Aside from these, the similarity was a strong suggestion of functional conservation. Finally, I observed very different hydrogen bonding and water molecule patterns inside the binding pocket, which was mainly due to several F to Y substitutions in that region. This might cause retinal to interact with adjacent residues in a subtly different way, and might lead to a different absorption peak since the environment of the RSB, that is, the binding pocket, determines the spectral characteristic of opsins (Fenno *et al.*, 2011).

Overall, I observed that QTY-designed OPN2 had relatively similar behavior to the native variant despite certain small differences caused by decreased hydrophobicity. The function of OPN2 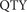 awaits to be verified by experiments.

### Future scopes and potential applications

The findings of this study indicate that the QTY code could be used as a robust tool to design water-soluble retinylidene proteins.

This could, in the first place, facilitate the study of these proteins. The water solubility of QTY analogues makes it easier to purify and investigate proteins, especially those such as RGR and RRH, which have not been studied much but may have considerable clinical relevance. The water solubility also reduces the difficulty in recording the different phases of the photocycle, in comparison with native membrane opsins. Another application may be the rapid design of new optogenetic tools.

Furthermore, water-soluble QTY opsins could be useful clinically, since they could be delivered and/or expressed in the eye without forming aggregates and precipitating. It is not unreasonable to hypothesize that QTY opsins can still pass through the photocycle and interact with downstream signaling proteins. In light of this, QTY opsins may facilitate the optogenetic therapy of ophthalmological diseases (Sakai *et al.*, 2022), providing a potential pathway in restoring basic vision for those who have lost it.

Finally, water-soluble opsins have the potential to be harvested in mass and may be used to design new biomimetic light-sensing systems, which may have applications in bioengineering.

## Conclusion

In this study, I selected 12 retinylidene proteins, including 9 human opsins (OPN1MW, OPN1LW, OPN1SW, OPN2, OPN3, OPN4, OPN5, RGR, and RRH) and 3 microbial opsins (BACR, BACH, and ChR2), that had clinical implications and various potential applications. I applied the QTY code to convert the hydrophobic transmembrane alpha helices into hydrophilic ones and thus create water-soluble QTY analogues of the proteins. Then, I used AlphaFold3 to predict the structures of the native proteins as well as the QTY analogues, superposed them, and found that the structure of the proteins was well conserved despite substantial residue substitutions. I also inspected the surface hydrophobicity of the proteins and found a great decrease in hydrophobic patches on the transmembrane surface. Next, I chose a representative retinylidene protein, OPN2, and employed molecular dynamics simulations to investigate the behavior of the native and QTY analogue of OPN2. I found that the QTY analogue was capable of conformational change and effective protein–ligand interaction. These findings revealed that QTY-designed OPN2 and retinylidene proteins in general might have conserved structure and function. I believe that these water-soluble QTY variants may have potential in research, therapeutic treatments, and bioengineering.

## Supporting information

Pan supplementary material Pan supplementary material

## Data Availability

The data for the AlphaFold3-predicted structures and MD simulation setting files and commands can be found on Zenodo (https://doi.org/10.5281/zenodo.15377505).

## Supplementary material

The supplementary material for this article can be found at http://doi.org/10.1017/qrd.2025.10009.
