# Supplementary material for: A structural and functional bioinformatics study of QTY-designed retinylidene proteins
Source: QRB Discov. 2025 Jul 14;6:e20. doi: 10.1017/qrd.2025.10009 (PMC12361693; doi:10.1017/qrd.2025.10009)

Supplementary Information

A STRUCTURAL AND FUNCTIONAL BIOINFORMATICS STUDY OF QTY-DESIGNED RETINYLIDENE PROTEINS

Siqi Pan

Figure S1. The enlarged protein sequence alignments of 12 retinylidene proteins and their QTY analogs from Figure 1. The symbols | and ∗ indicate that amino acids are identical or different, respectively. Amino acids L, I/V, and F in TM (transmembrane) alpha helices (shown in blue above the sequences) are replaced with Q, T, and Y respectively. The variation in the TM domain ranges from 35.53% to 50.24% while the overall variation rate ranges from 15.48% to 30.04%. The alignments are **a)** OPN1MW *vs* OPN1MW^{QTY}, **b)** OPN1LW *vs* OPN1LW^{QTY}, **c)** OPN1SW *vs* OPN1SW^{QTY}, **d)** OPN2 *vs* OPN2^{QTY}, **e)** OPN3 *vs* OPN3^{QTY}, **f)** OPN4 *vs* OPN4^{QTY}, **g)** OPN5 *vs* OPN5^{QTY}, **h)** RGR *vs* RGR^{QTY}, **i)** RRH *vs* RRH^{QTY}, **j)** BACR *vs* BACR^{QTY}, **k)** BACH *vs* BACH^{QTY}, **l)** ChR2 *vs* ChR2^{QTY}.

a) OPN1MW *vs* OPN1MW^{QTY}

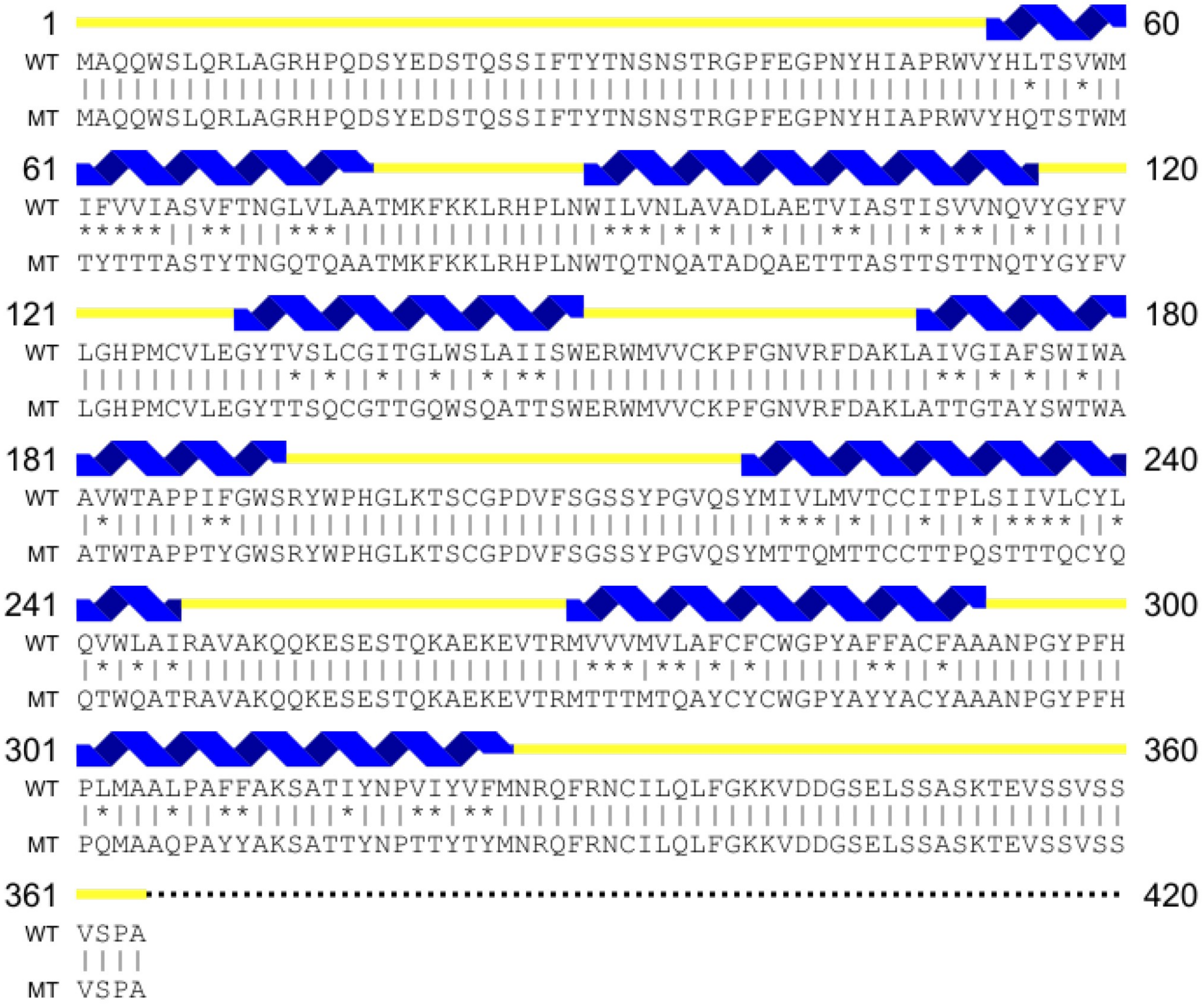

b) OPN1LW *vs* OPN1LW^{QTY}

```
1                                                                  60
WT  MAQQWSLQRLAGRHPQDSYEDSTQSSIFTYTNSNSTRGPFEGPNYHIAPRWVYHLTSVWM
    ||||||||||||||||||||||||||||||||||||||||||||||||||||||*||*||
MT  MAQQWSLQRLAGRHPQDSYEDSTQSSIFTYTNSNSTRGPFEGPNYHIAPRWVYHQTSTWM

61                                                                120
WT  IFVVTASVFTNGLVLAATMKFKKLRHPLNWILVNLAVADLAETVIASTISIVNQVSGYFV
    ****|||**|||***|||||||||||||||***|*|*||*|||**|||*|**||*|||||
MT  TYTTTASTYTNGQTQAATMKFKKLRHPLNWTQTNQATADQAETTTASTTSTTNQTSGYFV

121                                                               180
WT  LGHPMCVLEGYTVSLCGITGLWSLAIISWERWMVVCKPFGNVRFDAKLAIVGIAFSWIWA
    ||||||||||||*|*||*||*||*|**|||||||||||||||||||||||**|*|*||*||
MT  LGHPMCVLEGYTTSQCGTTGQWSQATTSWERWMVVCKPFGNVRFDAKLATTGTAYSWTWA

181                                                               240
WT  AVWTAPPIFGWSRYWPHGLKTSCGPDVFSGSSYPGVQSYMIVLMVTCCIIPLAIIMLCYL
    |*|||||**|||||||||||||||||||||||||||||||***|*|||**|*|**|*||*
MT  ATWTAPPTYGWSRYWPHGLKTSCGPDVFSGSSYPGVQSYMTTQMTTCCTTPQATTMQCYQ

241                                                               300
WT  QVWLAIRAVAKQQKESESTQKAEKEVTRMVVVMIFAYCVCWGPYTFFACFAAANPGYAFH
    |*|*|*|||||||||||||||||||||||***|**|||*||||||**||*||||||||||
MT  QTWQATRAVAKQQKESESTQKAEKEVTRMTTTMTYAYCTCWGPYTYYACYAAANPGYAFH

301                                                               360
WT  PLMAALPAYFAKSATIYNPVIYVFMNRQFRNCILQLFGKKVDDGSELSSASKTEVSSVSS
    |*|||*|||*|||||*|||**|**||||||||||||||||||||||||||||||||||||
MT  PQMAAQPAYYAKSATTYNPTTYTYMNRQFRNCILQLFGKKVDDGSELSSASKTEVSSVSS

361                                                               420
WT  VSPA
    ||||
MT  VSPA
```

c) OPN1SW *vs* OPN1SW^{QTY}

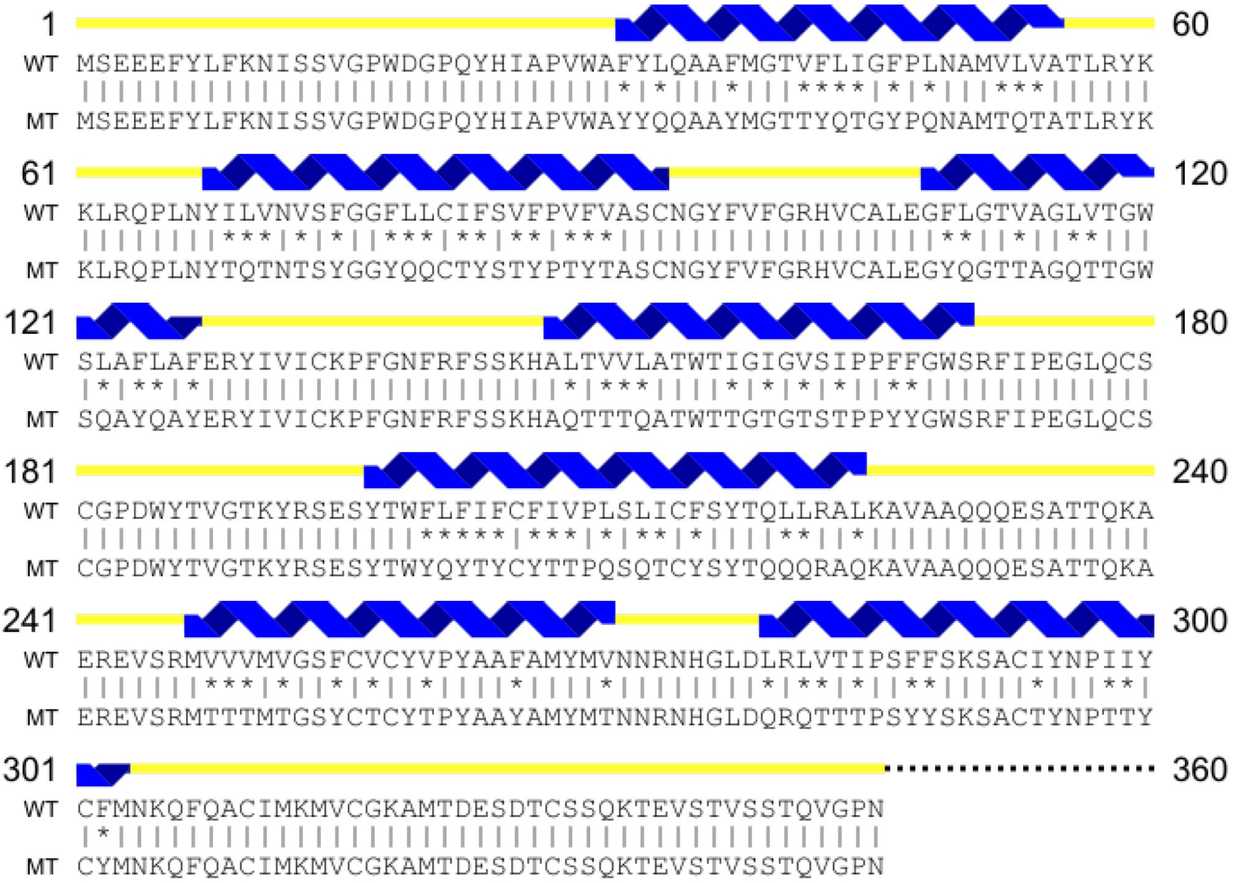

d) OPN2 *vs* OPN2^{QTY}

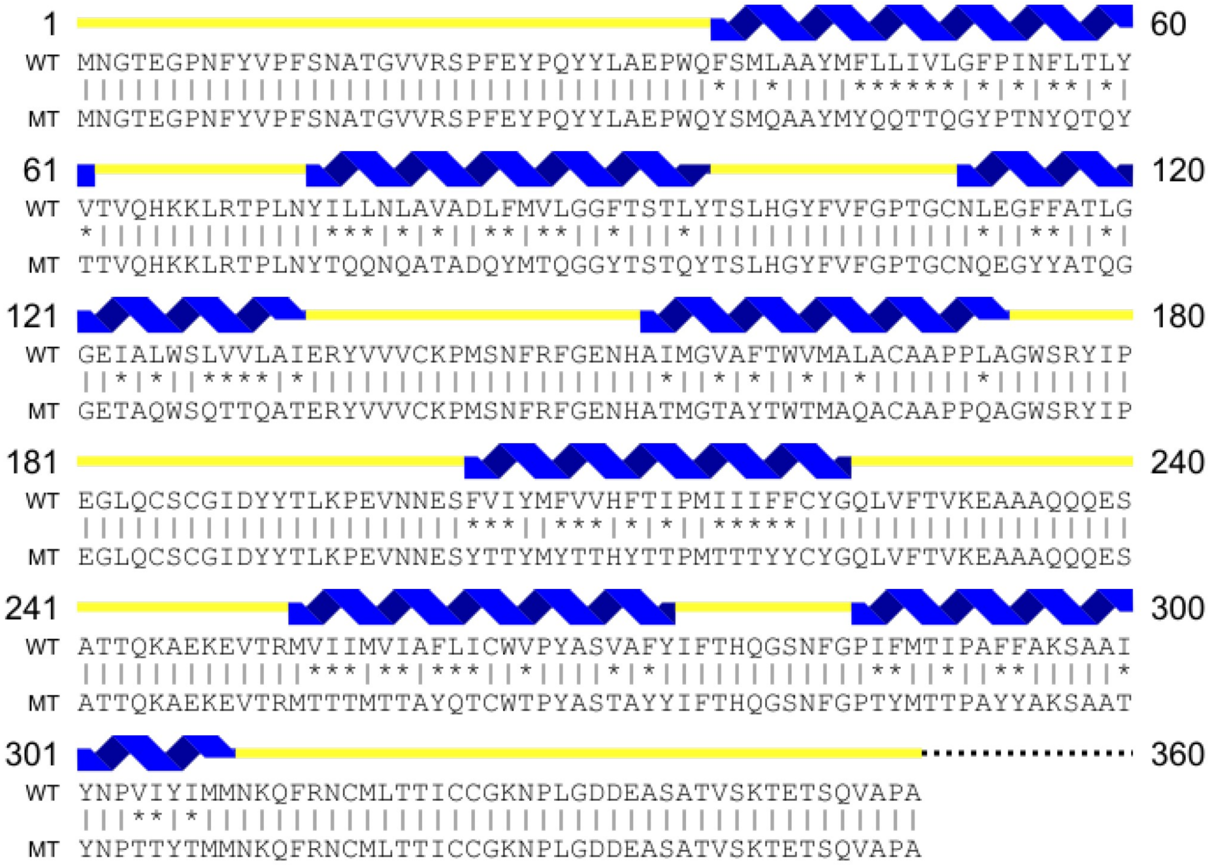

e) OPN3 *vs* OPN3^{QTY}

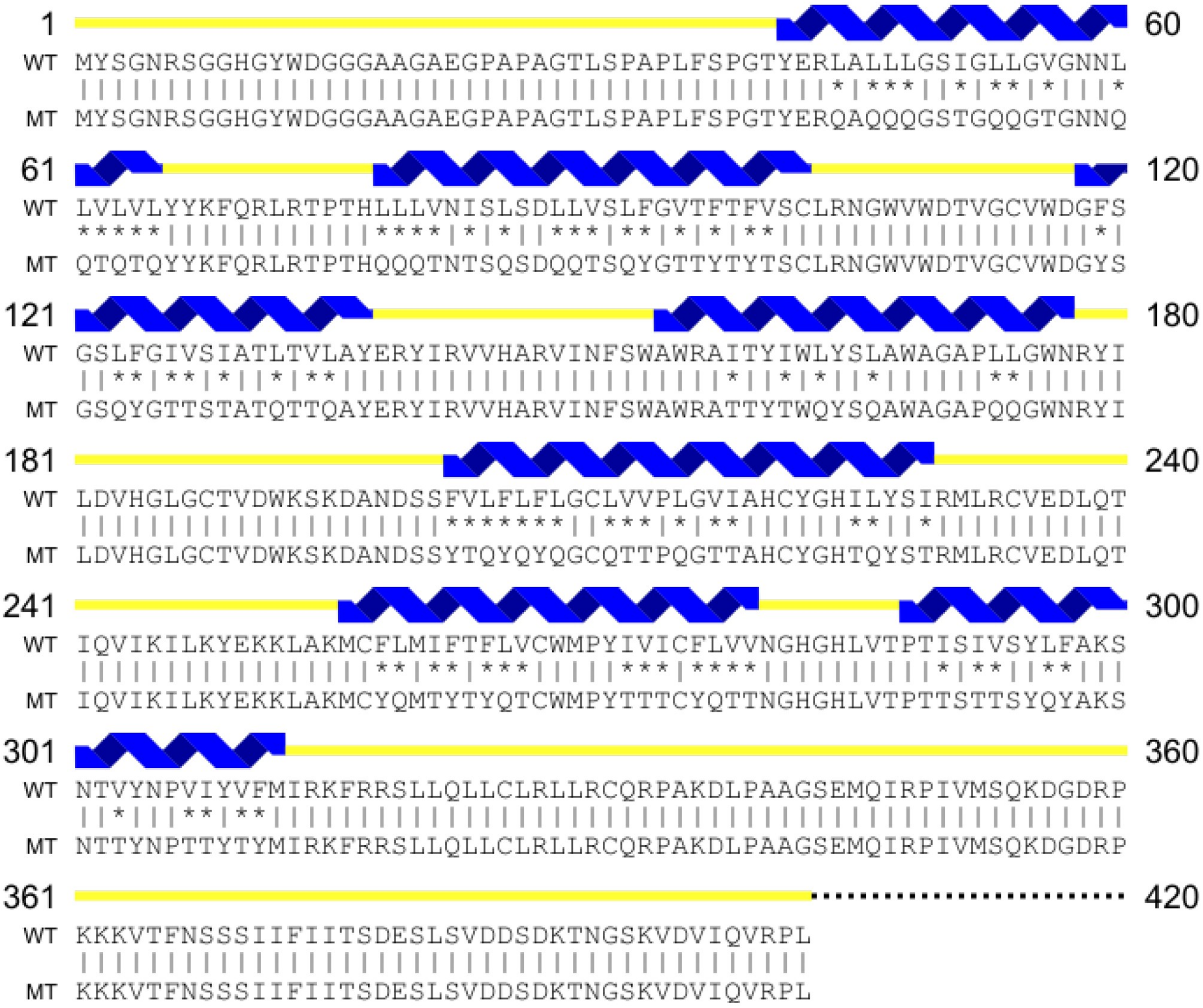

f) OPN4 *vs* OPN4^{QTY}

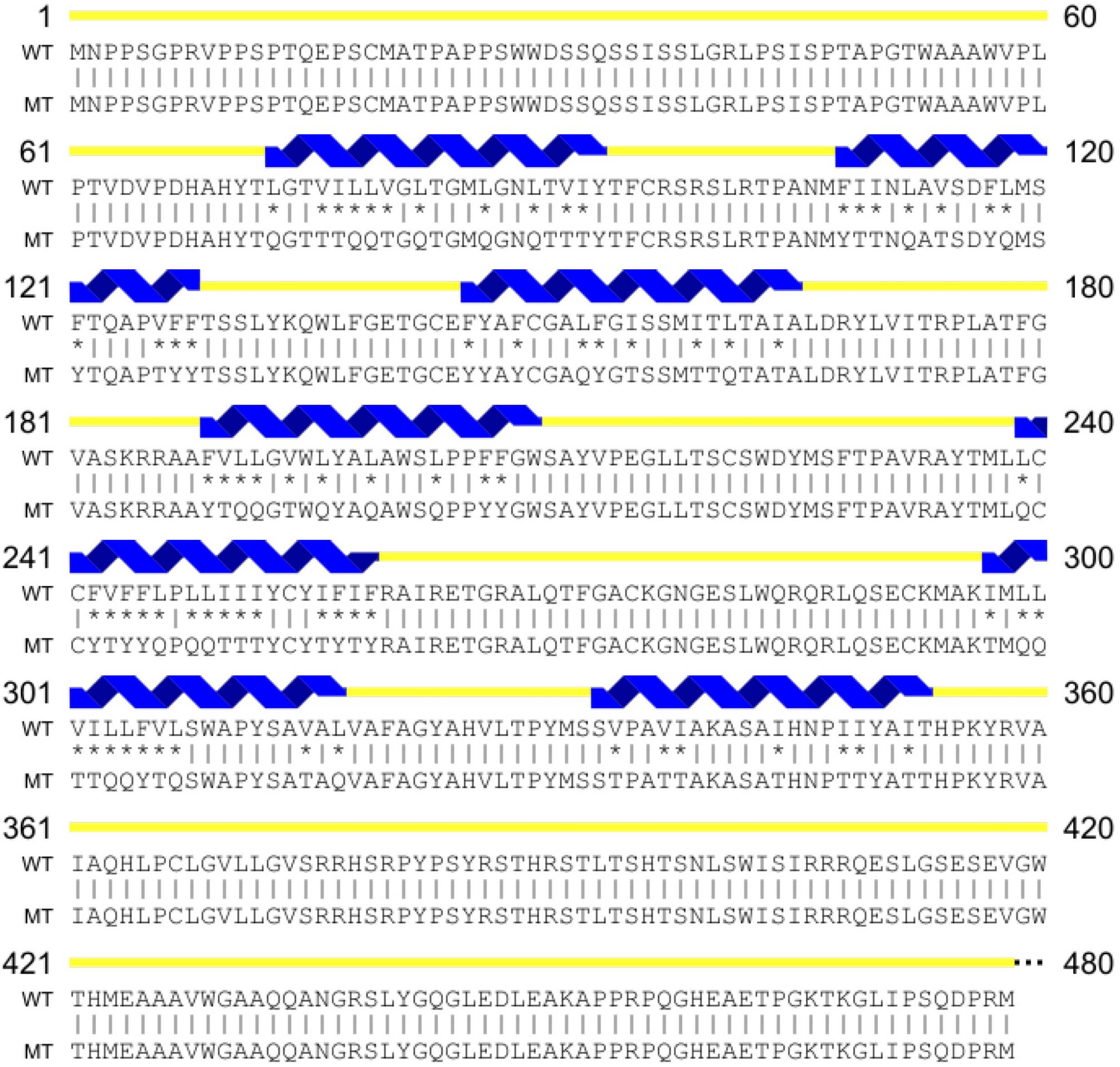

g) OPN5 *vs* OPN5^{QTY}

```
1                                                                 60
WT MALNHTALPQDERLPHYLRDGDPFASKLSWEADLVAGFYLTIIGILSTFGNGYVLYMSSR
   |||||||||||||||||||||||||||||||||**||*|*|**|**||*||||*||||||
MT MALNHTALPQDERLPHYLRDGDPFASKLSWEADQTAGYYQTTTGTQSTYGNGYTLYMSSR

61                                                               120
WT RKKKLRPAEIMTINLAVCDLGISVVGKPFTIISCFCHRWVFGWIGCRWYGWAGFFFGCGS
   ||||||||||||||*|*||*|*|**|||*|**||*||||||||||||||||||***||||
MT RKKKLRPAEIMTINQATCDQGTSTTGKPYTTTSCYCHRWVFGWIGCRWYGWAGYYYGCGS

121                                                              180
WT LITMTAVSLDRYLKICYLSYGVWLKRKHAYICLAAIWAYASFWTTMPLVGLGDYVPEPFG
   **||||*|*|||||||||||||||||||||*|*||*|||||*|||||**|*|||||||||
MT QTTMTATSQDRYLKICYLSYGVWLKRKHAYTCQAATWAYASYWTTMPQTGQGDYVPEPFG

181                                                              240
WT TSCTLDWWLAQASVGGQVFILNILFFCLLLPTAVIVFSYVKIIAKVKSSSKEVAHFDSRI
   |||||||||||||||||****|****|***|||****|||||||||||||||||||||||
MT TSCTLDWWLAQASVGGQTYTQNTQYYCQQQPTATTTYSYVKIIAKVKSSSKEVAHFDSRI

241                                                              300
WT HSSHVLEMKLTKVAMLICAGFLIAWIPYAVVSVWSAFGRPDSIPIQLSVVPTLLAKSAAM
   ||||||||||||*||**|||***||*|||**|*|||||||||||||||**||**||||||
MT HSSHVLEMKLTKTAMQTCAGYQTAWTPYATTSTWSAFGRPDSIPIQLSTTPTQQAKSAAM

301                                                              360
WT YNPIIYQVIDYKFACCQTGGLKATKKKSLEGFRLHTVTTVRKSSAVLEIHEEWE
   |||**||**|||||||||||||||||||||||||||||||||||||||||||||
MT YNPTTYQTTDYKFACCQTGGLKATKKKSLEGFRLHTVTTVRKSSAVLEIHEEWE
```

h) RGR *vs* RGR^{QTY}

```
1                                                                          60
WT  MAETSALPTGFGELEVLAVGMVLLVEALSGLSLNTLTIFSFCKTPELRTPCHLLVLSLAL
    |||||||||||||||**|*||****||*||*|*||*||||||||||||||||****|*|*
MT  MAETSALPTGFGELETQATGMTQQTEAQSGQSQNTQTIFSFCKTPELRTPCHQQTQSQAQ

61                                                                        120
WT  ADSGISLNALVAATSSLLRRWPYGSDGCQAHGFQGFVTALASICSSAAIAWGRYHHYCTR
    ||||*|*||**|||||||||||||||||||||*||**||*||*|||||*|||||||||||
MT  ADSGTSQNAQTAATSSLLRRWPYGSDGCQAHGYQGYTTAQASTCSSAATAWGRYHHYCTR

121                                                                       180
WT  SQLAWNSAVSLVLFVWLSSAFWAALPLLGWGHYDYEPLGTCCTLDYSKGDRNFTSFLFTM
    ||||||||||*****|*|||*|||*|**|||||||||||||||||||||||||||***||
MT  SQLAWNSAVSQTQYTWQSSAYWAAQPQQGWGHYDYEPLGTCCTLDYSKGDRNFTSYQYTM

181                                                                       240
WT  SFFNFAMPLFITITSYSLMEQKLGKSGHLQVNTTLPARTLLLGWGPYAILYLYAVIADVT
    |**|*|||***|*||||||||||||||||||||||||||***||||||**|*||**||*|
MT  SYYNYAMPQYTTTTSYSLMEQKLGKSGHLQVNTTLPARTQQQGWGPYATQYQYATTADTT

241                                                                       300
WT  SISPKLQMVPALIAKMVPTINAINYALGNEMVCRGIWQCLSPQKREKDRTK
    ||||||||*||**|||*||*||*|||*||||||||||||||||||||||||
MT  SISPKLQMTPAQTAKMTPTTNATNYAQGNEMVCRGIWQCLSPQKREKDRTK
```

i) RRH *vs* RRH^{QTY}

```
1                                                                  60
WT MLRNNLGNSSDSKNEDGSVFSQTEHNIVATYLIMAGMISIISNIIVLGIFIKYKELRTPT
   ||||||||||||||||||||||||||**|||**||||*|**||****|*|||||||||||
MT MLRNNLGNSSDSKNEDGSVFSQTEHNTTATYQTMAGMTSTTSNTTTQGTFIKYKELRTPT

61                                                                120
WT NAIIINLAVTDIGVSSIGYPMSAASDLYGSWKFGYAGCQVYAGLNIFFGMASIGLLTVVA
   ||***|*|*||*|*||*|||||||||*||||||||||||||||*|***||||*|**|**|
MT NATTTNQATTDTGTSSTGYPMSAASDQYGSWKFGYAGCQVYAGQNTYYGMASTGQQTTTA

121                                                               180
WT VDRYLTICLPDVGRRMTTNTYIGLILGAWINGLFWALMPIIGWASYAPDPTGATCTINWR
   *||||||||||||||||||||*|***|||*||**||*||**|||||||||||||||||||
MT TDRYLTICLPDVGRRMTTNTYTGQTQGAWTNGQYWAQMPTTGWASYAPDPTGATCTINWR

181                                                               240
WT KNDRSFVSYTMTVIAINFIVPLTVMFYCYYHVTLSIKHHTTSDCTESLNRDWSDQIDVTK
   ||||||||||||**|*|***|*|*|*|||||*||||||||||||||||||||||||||||
MT KNDRSFVSYTMTTTATNYTTPQTTMYYCYYHTTLSIKHHTTSDCTESLNRDWSDQIDVTK

241                                                               300
WT MSVIMICMFLVAWSPYSIVCLWASFGDPKKIPPPMAIIAPLFAKSSTFYNPCIYVVANKK
   ||**|*||***||||||**|*|||||||||||||||**||**|||||*||||*|**||||
MT MSTTMTCMYQTAWSPYSTTCQWASFGDPKKIPPPMATTAPQYAKSSTYYNPCTYTTANKK

301                                                               360
WT FRRAMLAMFKCQTHQTMPVTSILPMDVSQNPLASGRI
   |||||||||||||||||||||||||||||||||||||
MT FRRAMLAMFKCQTHQTMPVTSILPMDVSQNPLASGRI
```

j) BACR *vs* BACR^{QTY}

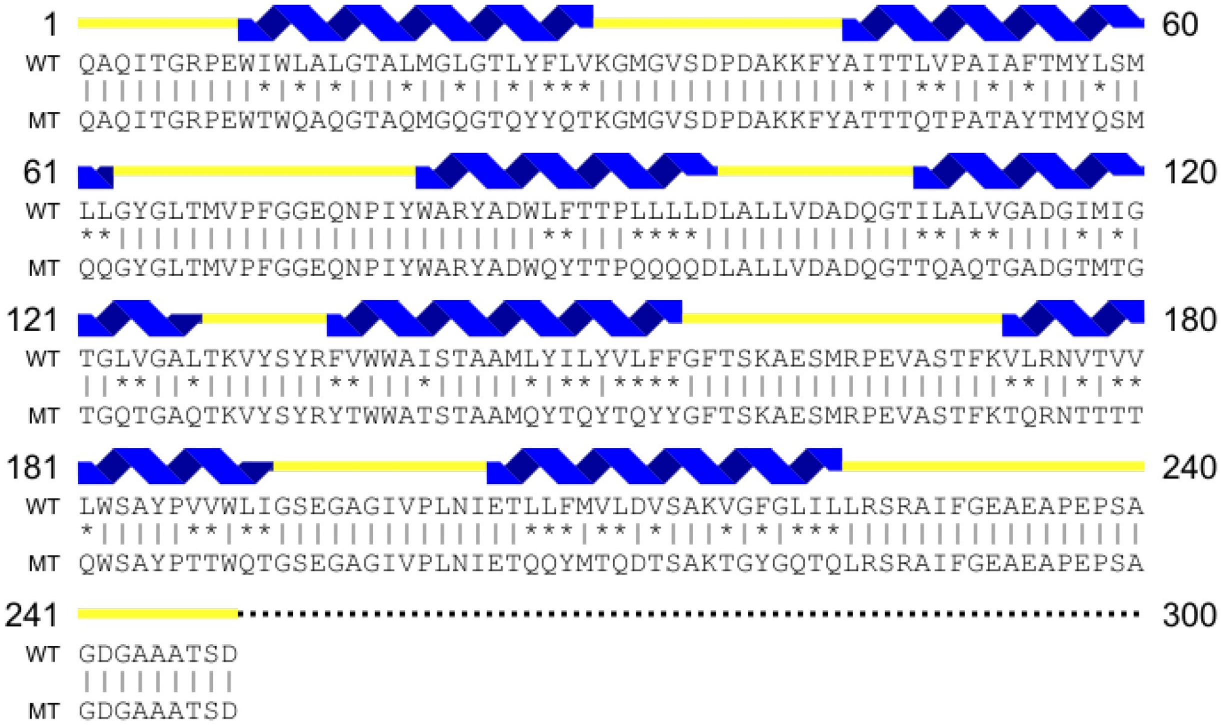

k) BACH *vs* BACH^{QTY}

```
1                                                                  60
WT AVRENALLSSSLWVNVALAGIAILVFVYMGRTIRPGRPRLIWGATLMIPLVSISSYLGLL
   ||||||**|||*|*|*|*||*|*****||||||||||||**||||*|*|**|*|||*|**
MT AVRENAQQSSSQWTNTAQAGTATQTYTYMGRTIRPGRPRQTWGATQMTPQTSTSSYQGQQ

61                                                                120
WT SGLTVGMIEMPAGHALAGEMVRSQWGRYLTWALSTPMILLALGLLADVDLGSLFTVIAAD
   ||||||||||||||||||||||||||||*|||*||||***|*|**||||*||**|**|||
MT SGLTVGMIEMPAGHALAGEMVRSQWGRYQTWAQSTPMTQQAQGQQADVDQGSQYTTTAAD

121                                                               180
WT IGMCVTGLAAAMTTSALLFRWAFYAISCAFFVVVLSALVTDWAASASSAGTAEIFDTLRV
   *|||*||*||||||||***|||*||*|||******||**||||||||||||||**||*|*
MT TGMCTTGQAAAMTTSAQQYRWAYYATSCAYYTTTQSAQTTDWAASASSAGTAETYDTQRT

181                                                               240
WT LTVVLWLGYPIVWAVGVEGLALVQSVGVTSWAYSVLDVFAKYVFAFILLRWVANNERTVA
   *|***|*|||**||*||||||||||*|*||||||**|**|||**|****||*||||||||
MT QTTTQWQGYPTTWATGVEGLALVQSTGTTSWAYSTQDTYAKYTYAYTQQRWTANNERTVA

241                                                               300
WT VAGQTLGTMSSDD
   |||||||||||||
MT VAGQTLGTMSSDD
```

I) ChR2 *vs* ChR2^{QTY}

```
1                                                                  60
WT MDYGGALSAVGRELLFVTNPVVVNGSVLVPEDQCYCAGWIESRGTNGAQTASNVLQWLAA
   |||||||||||||||||||||||||||||||||||||||||||||||||||||**||*||
MT MDYGGALSAVGRELLFVTNPVVVNGSVLVPEDQCYCAGWIESRGTNGAQTASNTQQWQAA

61                                                                120
WT GFSILLLMFYAYQTWKSTCGWEEIYVCAIEMVKVILEFFFEFKNPSMLYLATGHRVQWLR
   |*|****|*||||||||||||||*|*||*||*|***|***||||||||||||||||||*|
MT GYSTQQQMYYAYQTWKSTCGWEETYTCATEMTKTTQEYYYEFKNPSMLYLATGHRVQWQR

121                                                               180
WT YAEWLLTCPVILIHLSNLTGLSNDYSRRTMGLLVSDIGTIVWGATSAMATGYVKVIFFCL
   ||||**|||****|*||||||||||||||||***||*||**|||||||||||*|****|*
MT YAEWQQTCPTTQTHQSNLTGLSNDYSRRTMGQQTSDTGTTTWGATSAMATGYTKTTYYCQ

181                                                               240
WT GLCYGANTFFHAAKAYIEGYHTVPKGRCRQVVTGMAWLFFVSWGMFPILFILGPEGFGVL
   |*||||||**||||||*|||||*|||||||**|||||****||||*|*****||||||||
MT GQCYGANTYYHAAKAYTEGYHTTPKGRCRQTTTGMAWQYYTSWGMYPTQYTQGPEGFGVL

241                                                               300
WT SVYGSTVGHTIIDLMSKNCWGLLGHYLRVLIHEHILIHGDIRKTTKLNIGGTEIEVETLV
   |*||||*|||**|*|||||||**|||*|***|||***|||||||||||||||||||||||
MT STYGSTTGHTTTDQMSKNCWGQQGHYQRTQTHEHTQTHGDIRKTTKLNIGGTEIEVETLV

301                                                               360
WT EDEAEAGAVNKGTGK
   |||||||||||||||
MT EDEAEAGAVNKGTGK
```

Figure S2. AlphaFold3 prediction accuracy: plDDT, PAE, ipTM, and pTM scores. The scores are displayed for the following proteins: **a)** OPN1MW^{QTY}, **b)** OPN1LW^{QTY}, **c)** OPN1SW^{QTY}, **d)** OPN2^{QTY}, **e)** OPN3^{QTY}, **f)** OPN4^{QTY}, **g)** OPN5^{QTY}, **h)** RGR^{QTY}, **i)** RRH^{QTY}, **j)** BACR^{QTY} monomer, **k)** BACH^{QTY} monomer, **l)** ChR2^{QTY} monomer, **m)** BACR^{QTY} trimer, **n)** BACH^{QTY} trimer, **o)** ChR2^{QTY} dimer.

a) OPN1MW^{QTY}

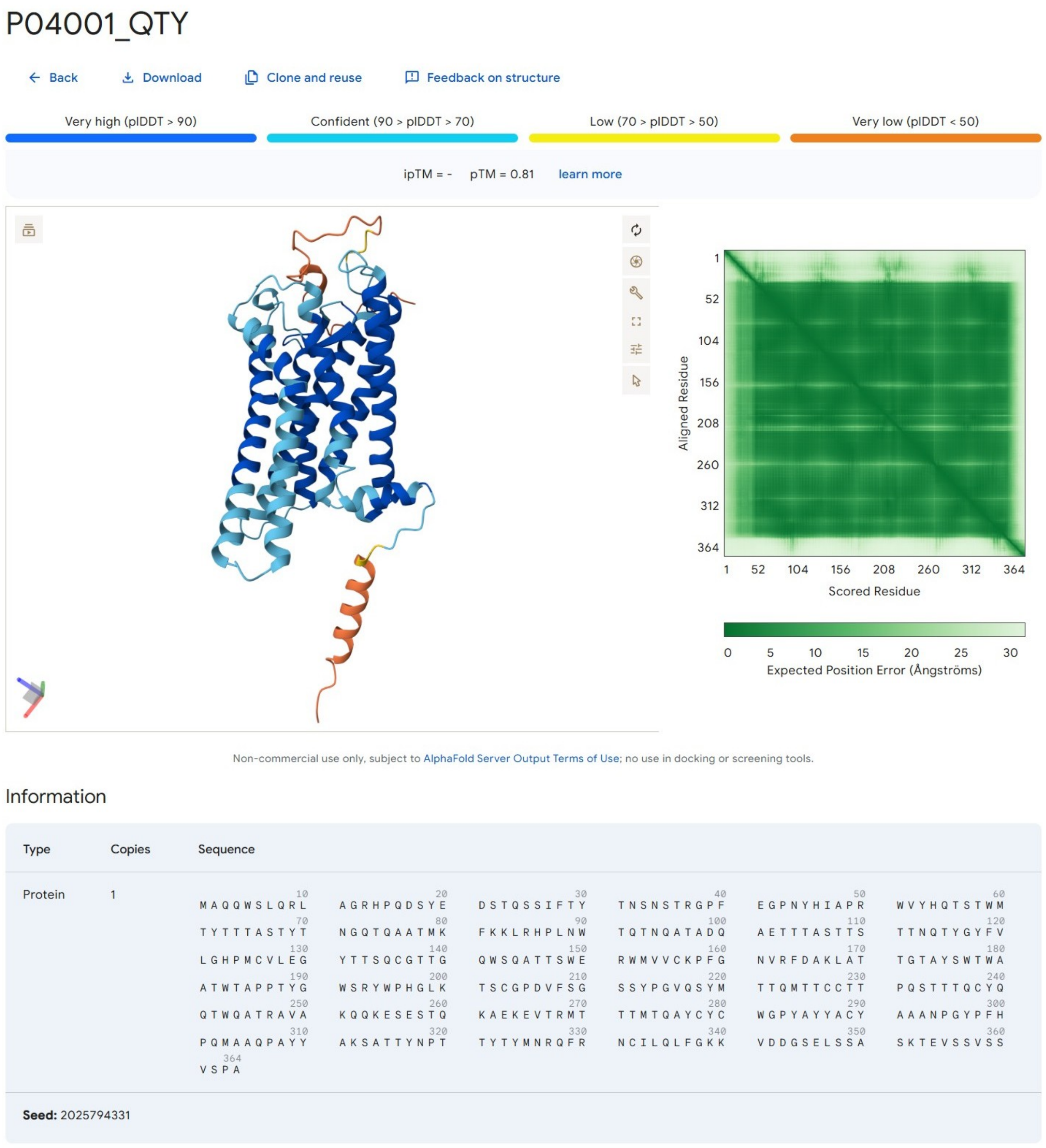

b) OPN1LW^{QTY}

P04000_QTY

← Back   Download   Clone and reuse   Feedback on structure

Very high (pIDDT > 90)	Confident (90 > pIDDT > 70)	Low (70 > pIDDT > 50)	Very low (pIDDT < 50)

ipTM = -   pTM = 0.82   learn more

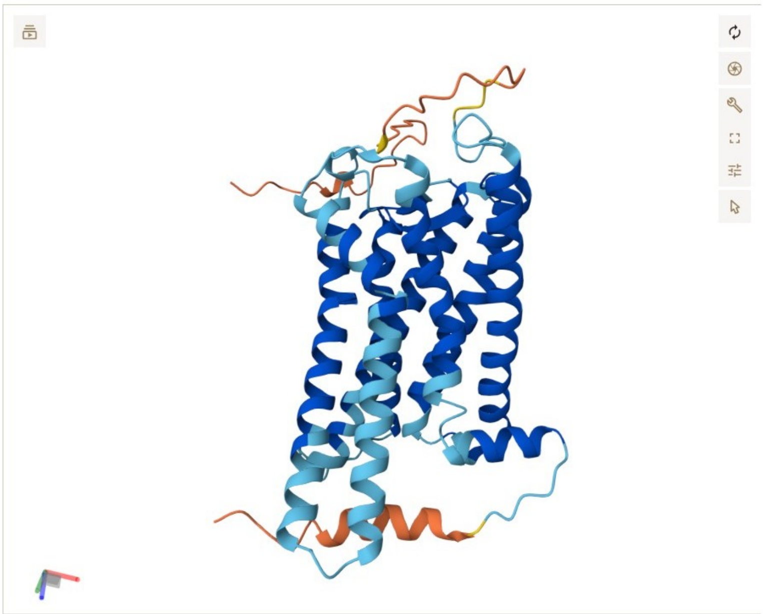

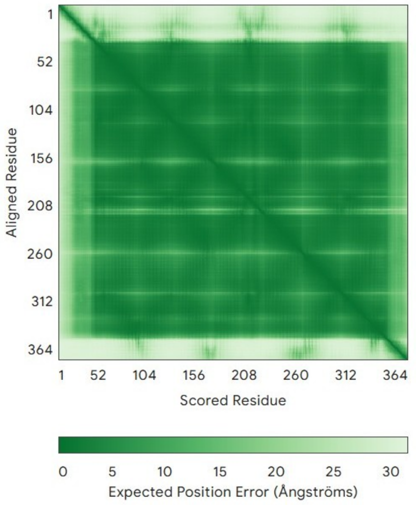

Non-commercial use only, subject to AlphaFold Server Output Terms of Use; no use in docking or screening tools.

Information

Type	Copies	Sequence
Protein	1	10 MAQQWSLQRL 20 AGRHPQDSYE 30 DSTQSSIFTY 40 TNSNSTRGPF 50 EGPNYHIAPR 60 WVYHQTSTWM<br>70 TYTTTASTYT 80 NGQTQAATMK 90 FKKLRHPLNW 100 TQTNQATADQ 110 AETTTASTTS 120 TTNQTSGYFV<br>130 LGHPMCVLEG 140 YTTSQCGTTG 150 QWSQATTSWE 160 RWMVVCKPFG 170 NVRFDAKLAT 180 TGTAYSWTWA<br>190 ATWTAPPTYG 200 WSRYWPHGLK 210 TSCGPDVFSG 220 SSYPGVQSYM 230 TTQMTTCCTT 240 PQATTMQCYQ<br>250 QTWQATRAVA 260 KQQKESESTQ 270 KAEKEVTRMT 280 TTMTYAYCTC 290 WGPYTYYACY 300 AAANPGYAFH<br>310 PQMAAQPAYY 320 AKSATTYNPT 330 TYTYMNRQFR 340 NCILQLFGKK 350 VDDGSELSSA 360 SKTEVSSVSS<br>364 VSPA

Seed: 621914961

c) OPN1SW^{QTY}

P03999_QTY

Back Download Clone and reuse Feedback on structure

Very high (pLDDT > 90) | Confident (90 > pLDDT > 70) | Low (70 > pLDDT > 50) | Very low (pLDDT < 50)

ipTM = -   pTM = 0.85   learn more

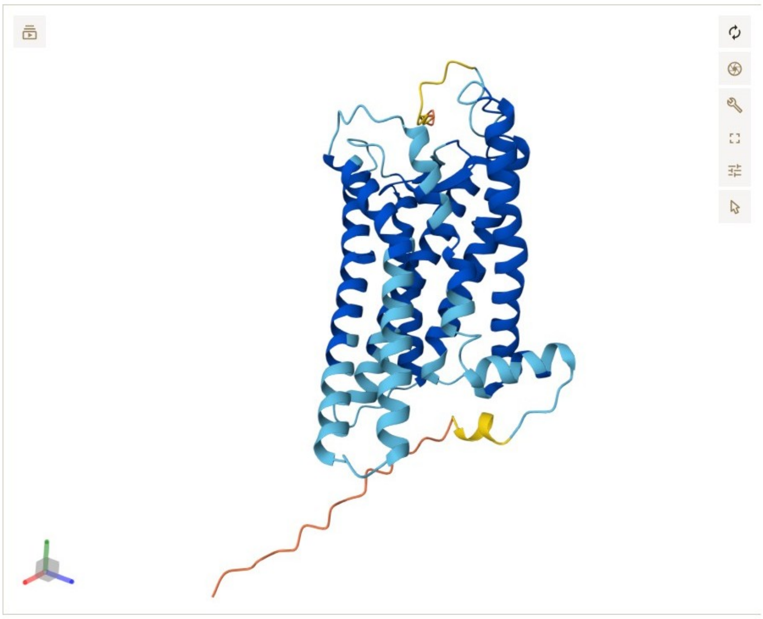

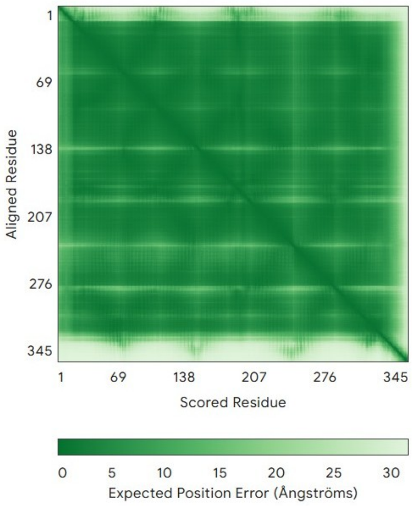

Non-commercial use only, subject to AlphaFold Server Output Terms of Use; no use in docking or screening tools.

Information

Type	Copies	Sequence
Protein	1	MSEEEFYLFK (10) NISSVGPWDG (20) PQYHIAPVWA (30) YYQQAAYMGT (40) TYQTGYPQNA (50) MTQTATLRYK (60)<br>KLRQPLNYTQ (70) TNTSYGGYQQ (80) CTYSTYPTYT (90) ASCNGYFVFG (100) RHVCALEGYQ (110) GTTAGQTTGW (120)<br>SQAYQAYERY (130) IVICKPFGNF (140) RFSSKHAQTT (150) TQATWTTGTG (160) TSTPPYYGWS (170) RFIPEGLQCS (180)<br>CGPDWYTVGT (190) KYRSESYTWY (200) QYTYCYTTPQ (210) SQTCYSYTQQ (220) QRAQKAVAAQ (230) QQESATTQKA (240)<br>EREVSRMTTT (250) MTGSYCTCYT (260) PYAAYAMYMT (270) NNRNHGLDQR (280) QTTTPSYYSK (290) SACTYNPTTY (300)<br>CYMNKQFQAC (310) IMKMVCGKAM (320) TDESDTCSSQ (330) KTEVSTVSST (340) QVGPN (345)

Seed: 1290598187

d) OPN2^{QTY}

P08100_QTY

← Back   Download   Clone and reuse   Feedback on structure

Very high (pIDDT > 90) | Confident (90 > pIDDT > 70) | Low (70 > pIDDT > 50) | Very low (pIDDT < 50)

ipTM = -   pTM = 0.87   learn more

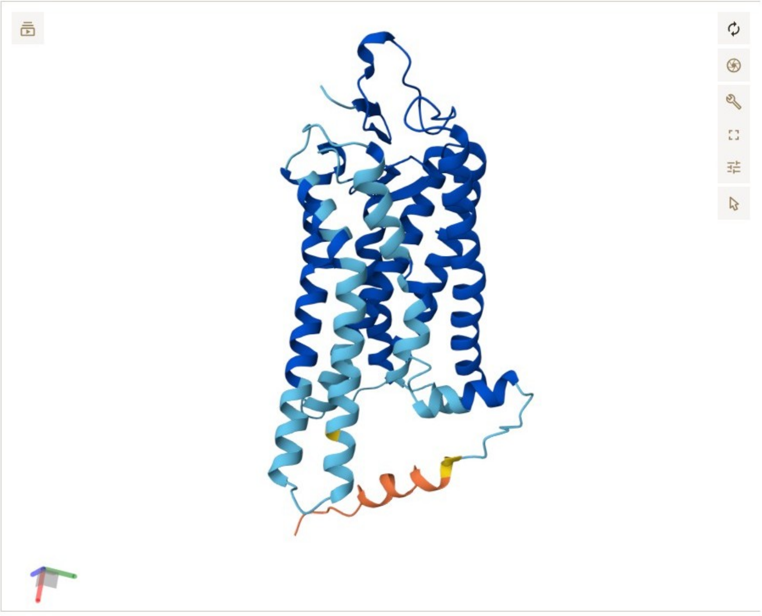

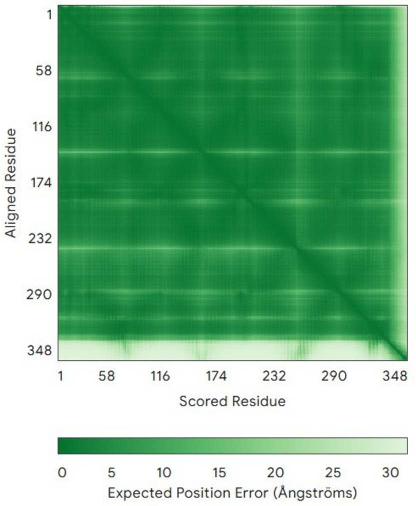

Non-commercial use only, subject to AlphaFold Server Output Terms of Use; no use in docking or screening tools.

Information

Type	Copies	Sequence
Protein	1	MNGTEGPNFY (10) VPFSNATGVV (20) RSPFEYPQYY (30) LAEPWQYSMQ (40) AAYMYQQTTQ (50) GYPTNYQTQY (60) TTVQHKKLRT (70) PLNYTQQNQA (80) TADQYMTQGG (90) YTSTQYTSLH (100) GYFVFGPTGC (110) NQEGYYATQG (120) GETAQWSQTT (130) QATERYVVVC (140) KPMSNFRFGE (150) NHATMGTAYT (160) WTMAQACAAP (170) PQAGWSRYIP (180) EGLQCSCGID (190) YYTLKPEVNN (200) ESYTTYMYTT (210) HYTTPMTTTY (220) YCYGQLVFTV (230) KEAAAQQQES (240) ATTQKAEKEV (250) TRMTTTMTTA (260) YQTCWTPYAS (270) TAYYIFTHQG (280) SNFGPTYMTT (290) PAYYAKSAAT (300) YNPTTYTMMN (310) KQFRNCMLTT (320) ICCGKNPLGD (330) DEASATVSKT (340) ETSQVAPA (348)

Seed: 1745886941

e) OPN3[QTY]

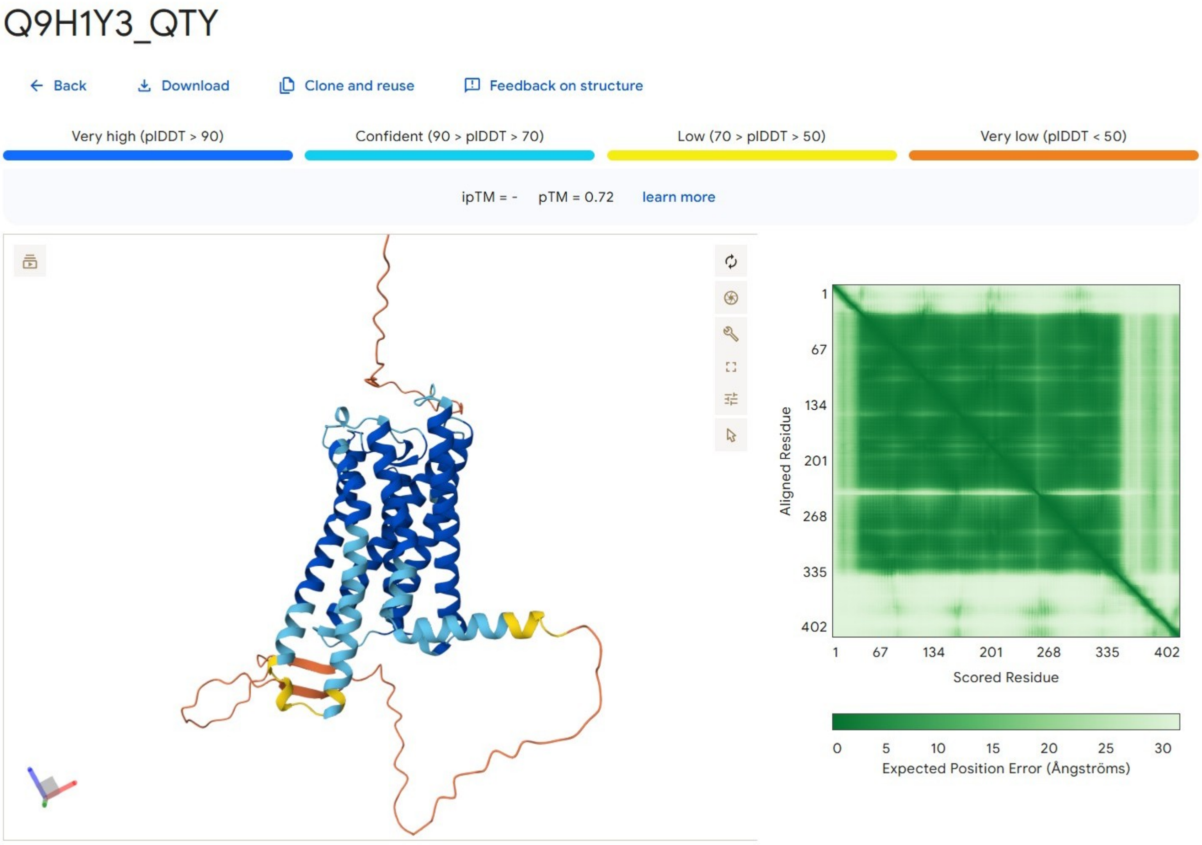

Non-commercial use only, subject to AlphaFold Server Output Terms of Use; no use in docking or screening tools.

Information

Type	Copies	Sequence
Protein	1	10 MYSGNRSGGH 20 GYWDGGGAAG 30 AEGPAPAGTL 40 SPAPLFSPGT 50 YERQAQQQGS 60 TGQQGTGNNQ<br>70 QTQTQYYKFQ 80 RLRTPTHQQQ 90 TNTSQSDQQT 100 SQYGTTYTYT 110 SCLRNGWVWD 120 TVGCVWDGYS<br>130 GSQYGTTSTA 140 TQTTQAYERY 150 IRVVHARVIN 160 FSWAWRATTY 170 TWQYSQAWAG 180 APQQGWNRYI<br>190 LDVHGLGCTV 200 DWKSKDANDS 210 SYTQYQYQGC 220 QTTPQGTTAH 230 CYGHTQYSTR 240 MLRCVEDLQT<br>250 IQVIKILKYE 260 KKLAKMCYQM 270 TYTYQTCWMP 280 YTTTCYQTTN 290 GHGHLVTPTT 300 STTSYQYAKS<br>310 NTTYNPTTYT 320 YMIRKFRRSL 330 LQLLCLRLLR 340 CQRPAKDLPA 350 AGSEMQIRPI 360 VMSQKDGDRP<br>370 KKKVTFNSSS 380 IIFIITSDES 390 LSVDDSDKTN 400 GSKVDVIQVR 402 PL

Seed: 666755378

f) OPN4^{QTY}

Q9UHM6_QTY

Back Download Clone and reuse Feedback on structure

Very high (pIDDT > 90) | Confident (90 > pIDDT > 70) | Low (70 > pIDDT > 50) | Very low (pIDDT < 50)

ipTM = -   pTM = 0.67   learn more

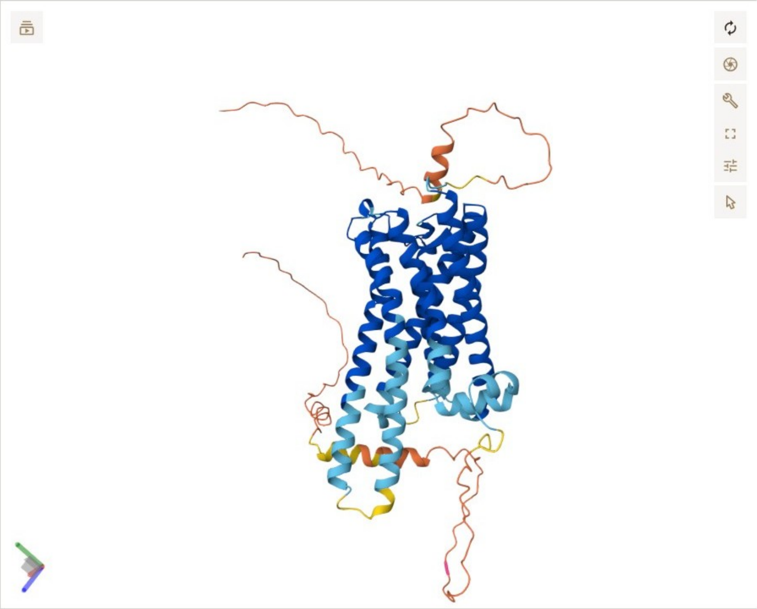

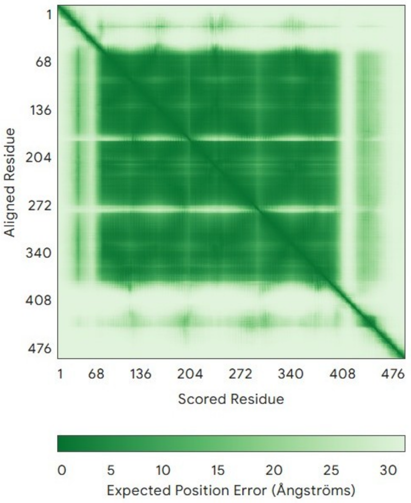

Non-commercial use only, subject to AlphaFold Server Output Terms of Use; no use in docking or screening tools.

Information

Type	Copies	Sequence
Protein	1	(see below)

MNPPSGPRVP 10	PSPTQEPSCM 20	ATPAPPSWWD 30	SSQSSISSLG 40	RLPSISPTAP 50	GTWAAAWVPL 60
PTVDVPDHAH 70	YTQGTTTQQT 80	GQTGMQGNQT 90	TTYTFCRSRS 100	LRTPANMYTT 110	NQATSDYQMS 120
YTQAPTYYTS 130	SLYKQWLFGE 140	TGCEYYAYCG 150	AQYGTSSMTT 160	QTATALDRYL 170	VITRPLATFG 180
VASKRRAAYT 190	QQGTWQYAQA 200	WSQPPYYGWS 210	AYVPEGLLTS 220	CSWDYMSFTP 230	AVRAYTMLQC 240
CYTYYQPQQT 250	TTYCYTYTYR 260	AIRETGRALQ 270	TFGACKGNGE 280	SLWQRQRLQS 290	ECKMAKTMQQ 300
TTQQYTQSWA 310	PYSATAQVAF 320	AGYAHVLTPY 330	MSSTPATTAK 340	ASATHNPTTY 350	ATTHPKYRVA 360
IAQHLPCLGV 370	LLGVSRRHSR 380	PYPSYRSTHR 390	STLTSHTSNL 400	SWISIRRRQE 410	SLGSESEVGW 420
THMEAAAVWG 430	AAQQANGRSL 440	YGQGLEDLEA 450	KAPPRPQGHE 460	AETPGKTKGL 470	IPSQDPRM 478

Seed: 1924211229

g) OPN5^{QTY}

Q6U736_QTY

Back Download Clone and reuse Feedback on structure

Very high (pIDDT > 90) | Confident (90 > pIDDT > 70) | Low (70 > pIDDT > 50) | Very low (pIDDT < 50)

ipTM = - pTM = 0.8 learn more

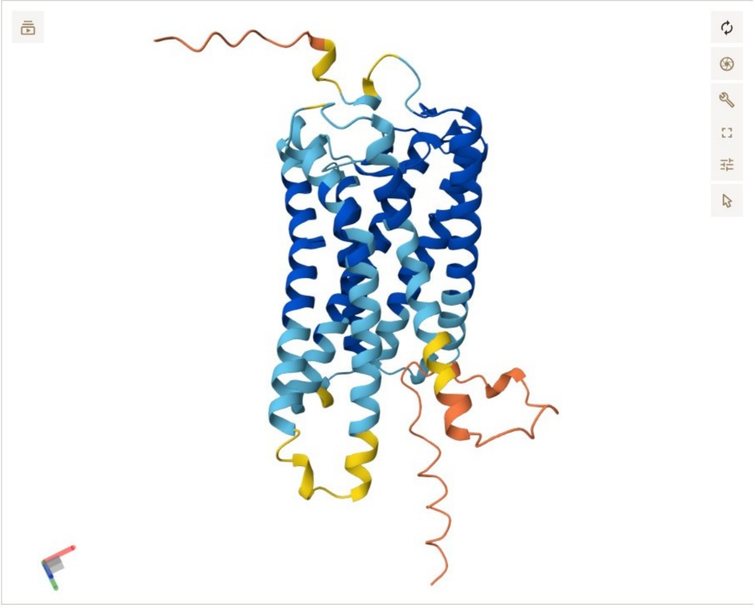

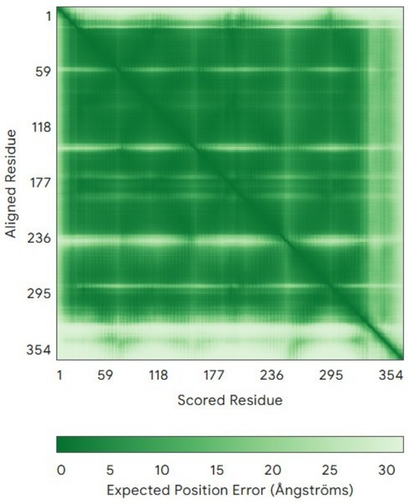

Non-commercial use only, subject to AlphaFold Server Output Terms of Use; no use in docking or screening tools.

Information

Type	Copies	Sequence
Protein	1	MALNHTALPQ DERLPHYLRD GDPFASKLSW EADQTAGYYQ TTTGTQSTYG NGYTLYMSSR RKKKLRPAEI MTINQATCDQ GTSTTGKPYT TTSCYCHRWV FGWIGCRWYG WAGYYYGCGS QTTMTATSQD RYLKICYLSY GVWLKRKHAY TCQAATWAYA SYWTTMPQTG QGDYVPEPFG TSCTLDWWLA QASVGGQTYT QNTQYYCQQQ PTATTTYSYV KIIAKVKSSS KEVAHFDSRI HSSHVLEMKL TKTAMQTCAG YQTAWTPYAT TSTWSAFGRP DSIPIQLSTT PTQQAKSAAM YNPTTYQTTD YKFACCQTGG LKATKKKSLE GFRLHTVTTV RKSSAVLEIH EEWE

Seed: 557682810

h) RGR^{QTY}

P47804_QTY

← Back Download Clone and reuse Feedback on structure

Very high (pIDDT > 90) | Confident (90 > pIDDT > 70) | Low (70 > pIDDT > 50) | Very low (pIDDT < 50)

ipTM = - pTM = 0.88 learn more

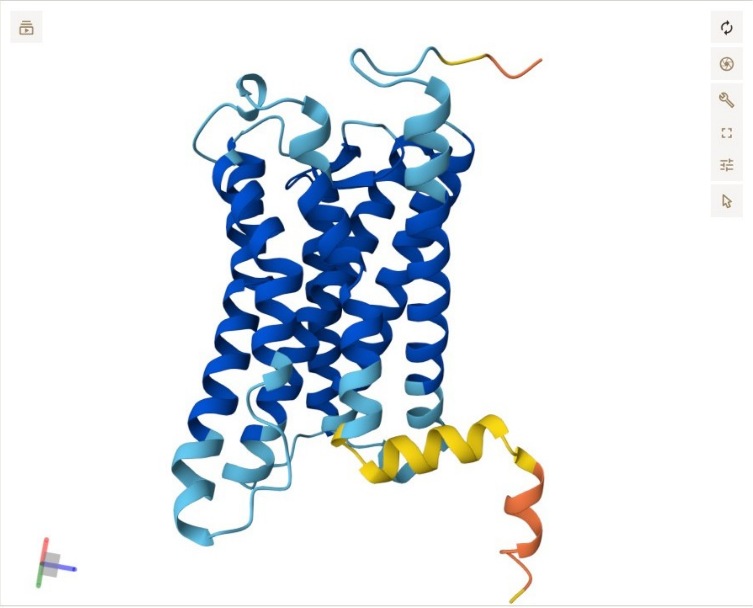

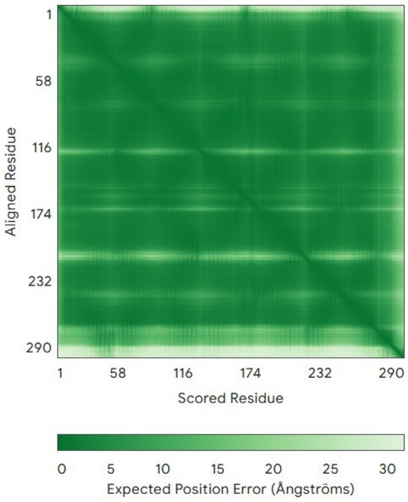

Non-commercial use only, subject to AlphaFold Server Output Terms of Use; no use in docking or screening tools.

Information

Type	Copies	Sequence
Protein	1	MAETSALPTG (10) FGELETQATG (20) MTQQTEAQSG (30) QSQNTQTIFS (40) FCKTPELRTP (50) CHQQTQSQAQ (60) ADSGTSQNAQ (70) TAATSSLLRR (80) WPYGSDGCQA (90) HGYQGYTTAQ (100) ASTCSSAATA (110) WGRYHHYCTR (120) SQLAWNSAVS (130) QTQYTWQSSA (140) YWAAQPQQGW (150) GHYDYEPLGT (160) CCTLDYSKGD (170) RNFTSYQYTM (180) SYYNYAMPQY (190) TTTTSYSLME (200) QKLGKSGHLQ (210) VNTTLPARTQ (220) QQGWGPYATQ (230) YQYATTADTT (240) SISPKLQMTP (250) AQTAKMTPTT (260) NATNYAQGNE (270) MVCRGIWQCL (280) SPQKREKDRT (290) K (291)

Seed: 938470342

i) RRH^{QTY}

O14718_QTY

Back Download Clone and reuse Feedback on structure

Very high (pIDDT > 90)	Confident (90 > pIDDT > 70)	Low (70 > pIDDT > 50)	Very low (pIDDT < 50)

ipTM = - pTM = 0.8 learn more

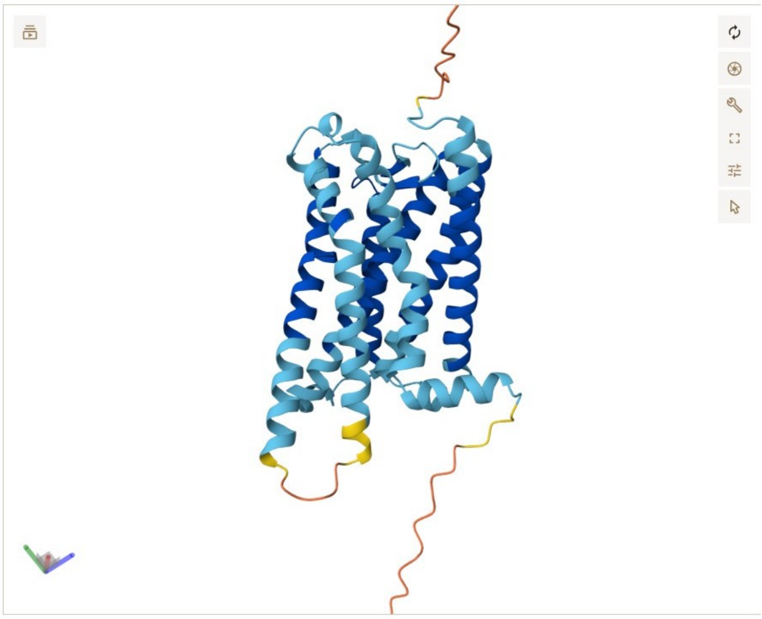

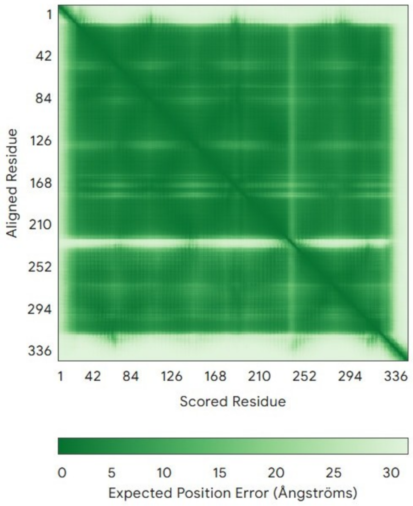

Non-commercial use only, subject to AlphaFold Server Output Terms of Use; no use in docking or screening tools.

Information

Type	Copies	Sequence
Protein	1	10 MLRNNLGNSS 20 DSKNEDGSVF 30 SQTEHNTTAT 40 YQTMAGMTST 50 TSNTTTQGTF 60 IKYKELRTPT<br>70 NATTTNQATT 80 DTGTSSTGYP 90 MSAASDQYGS 100 WKFGYAGCQV 110 YAGQNTYYGM 120 ASTGQQTTTA<br>130 TDRYLTICLP 140 DVGRRMTTNT 150 YTGQTQGAWT 160 NGQYWAQMPT 170 TGWASYAPDP 180 TGATCTINWR<br>190 KNDRSFVSYT 200 MTTTATNYTT 210 PQTTMYYCYY 220 HTTLSIKHHT 230 TSDCTESLNR 240 DWSDQIDVTK<br>250 MSTTMTCMYQ 260 TAWSPYSTTC 270 QWASFGDPKK 280 IPPPMATTAP 290 QYAKSSTYYN 300 PCTYTTANKK<br>310 FRRAMLAMFK 320 CQTHQTMPVT 330 SILPMDVSQN 337 PLASGRI

Seed: 1556700232

j) BACR^{QTY} monomer

P02945_QTY_monomer

Back   Download   Clone and reuse   Feedback on structure

Very high (pIDDT > 90)   Confident (90 > pIDDT > 70)   Low (70 > pIDDT > 50)   Very low (pIDDT < 50)

ipTM = -   pTM = 0.88   learn more

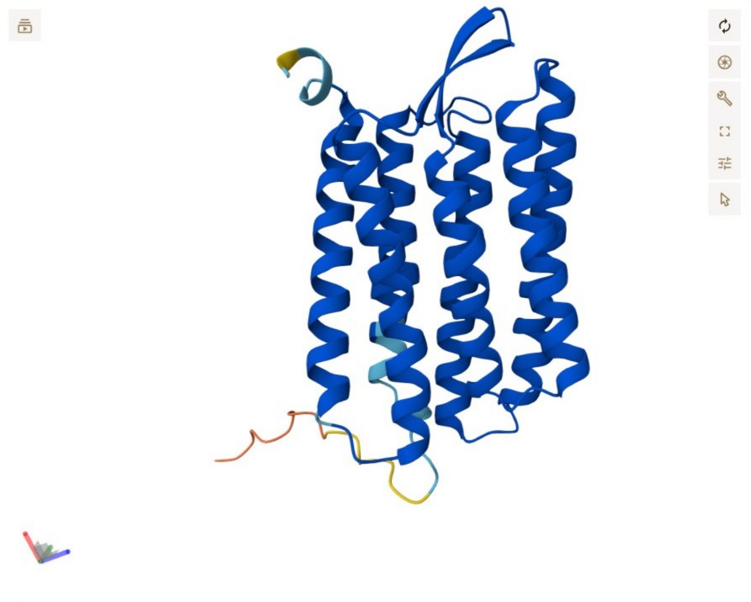

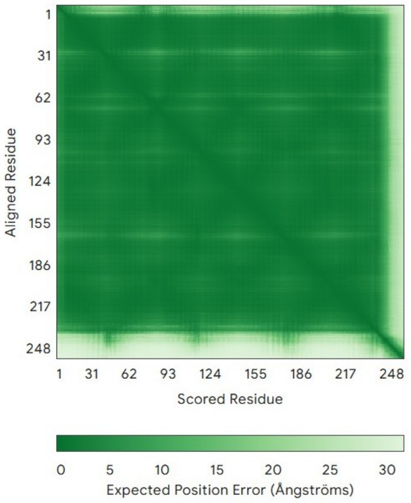

Non-commercial use only, subject to AlphaFold Server Output Terms of Use; no use in docking or screening tools.

Information

Type	Copies	Sequence
Protein	1	QAQITGRPEW (10) TWQAQGTAQM (20) GQGTQYYQTK (30) GMGVSDPDAK (40) KFYATTTQTP (50) ATAYTMYQSM (60) QQGYGLTMVP (70) FGGEQNPIYW (80) ARYADWQYTT (90) PQQQQDLALL (100) VDADQGTTQA (110) QTGADGTMTG (120) TGQTGAQTKV (130) YSYRYTWWAT (140) STAAMQYTQY (150) TQYYGFTSKA (160) ESMRPEVAST (170) FKTQRNTTTT (180) QWSAYPTTWQ (190) TGSEGAGIVP (200) LNIETQQYMT (210) QDTSAKTGYG (220) QTQLRSRAIF (230) GEAEAPEPSA (240) GDGAAATSD (249)

Seed: 265861460

k) BACH^{QTY} monomer

B0R2U4_QTY_monomer

← Back Download Clone and reuse Feedback on structure

Very high (pIDDT > 90)	Confident (90 > pIDDT > 70)	Low (70 > pIDDT > 50)	Very low (pIDDT < 50)

ipTM = - pTM = 0.89 learn more

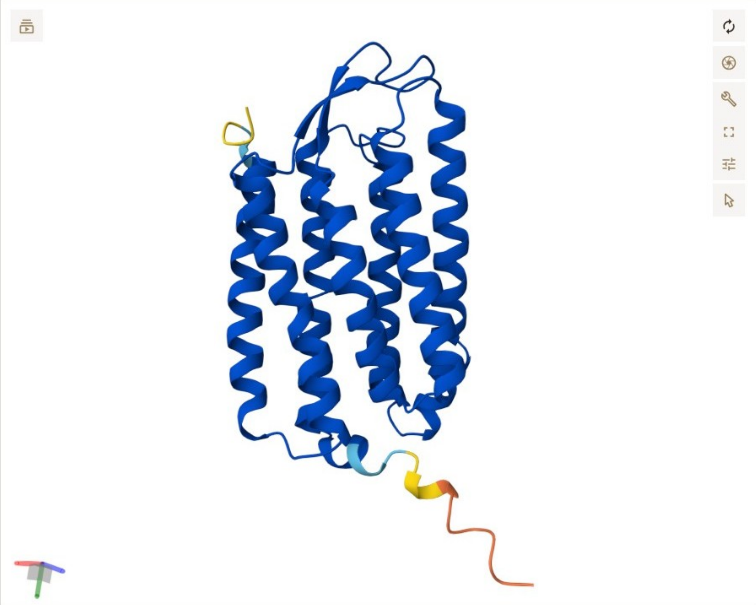

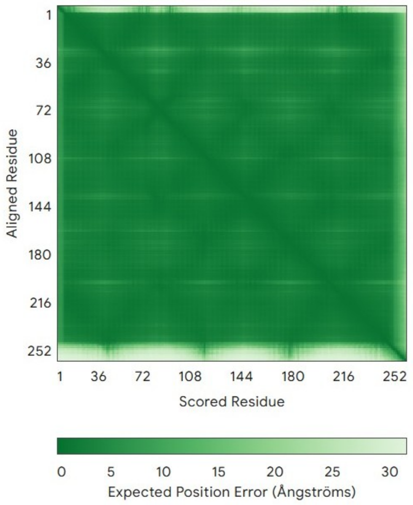

Non-commercial use only, subject to AlphaFold Server Output Terms of Use; no use in docking or screening tools.

Information

Type	Copies	Sequence
Protein	1	AVRENAQQSS(10) SQWTNTAQAG(20) TATQTYTYMG(30) RTIRPGRPRQ(40) TWGATQMTPQ(50) TSTSSYQGQQ(60) SGLTVGMIEM(70) PAGHALAGEM(80) VRSQWGRYQT(90) WAQSTPMTQQ(100) AQGQQADVDQ(110) GSQYTTTAAD(120) TGMCTTGQAA(130) AMTTSAQQYR(140) WAYYATSCAY(150) YTTTQSAQTT(160) DWAASASSAG(170) TAETYDTQRT(180) QTTTQWQGYP(190) TTWATGVEGL(200) ALVQSTGTTS(210) WAYSTQDTYA(220) KYTYAYTQQR(230) WTANNERTVA(240) VAGQTLGTMS(250) SDD(253)

Seed: 540778754

l) ChR2^{QTY} monomer

Q8RUT8_QTY_monomer

← Back   Download   Clone and reuse   Feedback on structure

Very high (pIDDT > 90)   Confident (90 > pIDDT > 70)   Low (70 > pIDDT > 50)   Very low (pIDDT < 50)

ipTM = -   pTM = 0.88   learn more

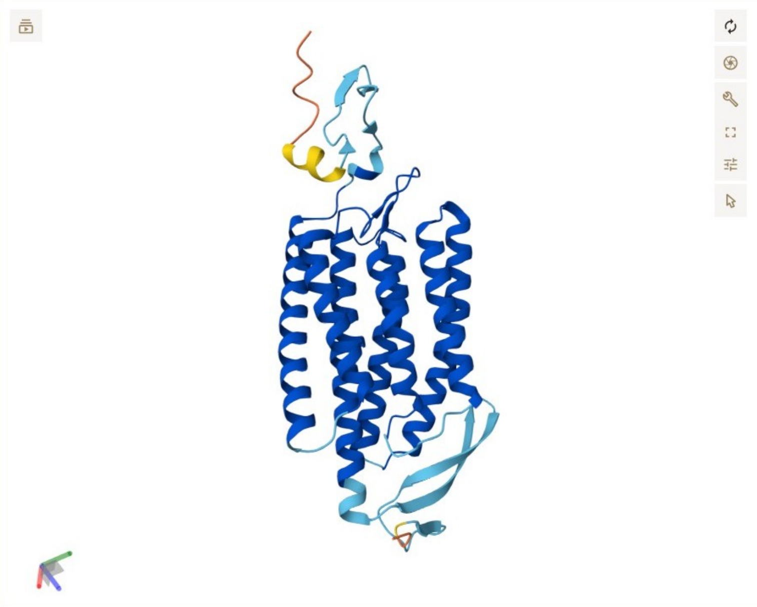

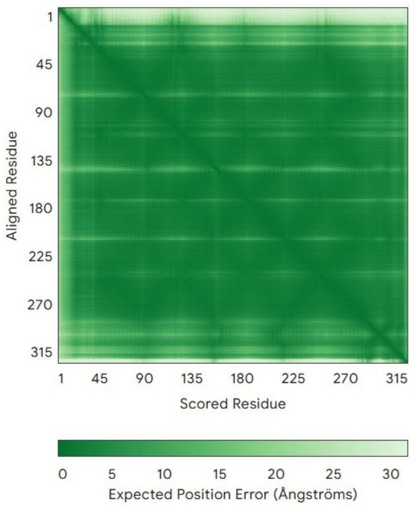

Non-commercial use only, subject to AlphaFold Server Output Terms of Use; no use in docking or screening tools.

Information

Type	Copies	Sequence
Protein	1	MDYGGALSAV GRELLFVTNP VVVNGSVLVP EDQCYCAGWI ESRGTNGAQT ASNTQQWQAA GYSTQQQMYY AYQTWKSTCG WEETYTCATE MTKTTQEYYY EFKNPSMLYL ATGHRVQWQR YAEWQQTCPT TQTHQSNLTG LSNDYSRRTM GQQTSDTGTT TWGATSAMAT GYTKTTYYCQ GQCYGANTYY HAAKAYTEGY HTTPKGRCRQ TTTGMAWQYY TSWGMYPTQY TQGPEGFGVL STYGSTTGHT TTDQMSKNCW GQQGHYQRTQ THEHTQTHGD IRKTTKLNIG GTEIEVETLV EDEAEAGAVN KGTGK

Seed: 746240196

m) BACR^{QTY} trimer

P02945_QTY_trimer

← Back   Download   Clone and reuse   Feedback on structure

Very high (pIDDT > 90)	Confident (90 > pIDDT > 70)	Low (70 > pIDDT > 50)	Very low (pIDDT < 50)

ipTM = 0.59   pTM = 0.65   learn more

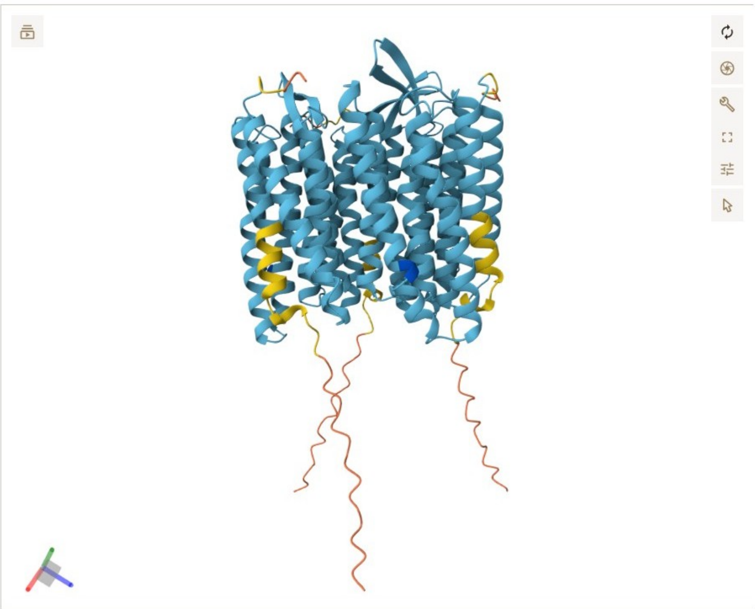

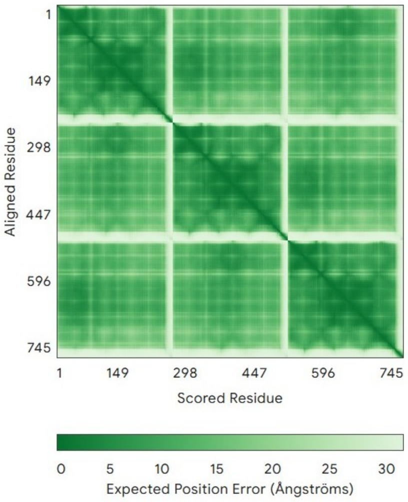

Non-commercial use only, subject to AlphaFold Server Output Terms of Use; no use in docking or screening tools.

Information

Type	Copies	Sequence
Protein	3	QAQITGRPEW TWQAQGTAQM GQGTQYYQTK GMGVSDPDAK KFYATTTQTP ATAYTMYQSM QQGYGLTMVP FGGEQNPIYW ARYADWQYTT PQQQQDLALL VDADQGTTQA QTGADGTMTG TGQTGAQTKV YSYRYTWWAT STAAMQYTQY TQYYGFTSKA ESMRPEVAST FKTQRNTTTT QWSAYPTTWQ TGSEGAGIVP LNIETQQYMT QDTSAKTGYG QTQLRSRAIF GEAEAPEPSA GDGAAATSD

Seed: 1970661198

n) BACH^{QTY} trimer

B0R2U4_QTY_trimer

← Back   Download   Clone and reuse   Feedback on structure

Very high (pIDDT > 90)	Confident (90 > pIDDT > 70)	Low (70 > pIDDT > 50)	Very low (pIDDT < 50)

ipTM = 0.38   pTM = 0.52   learn more

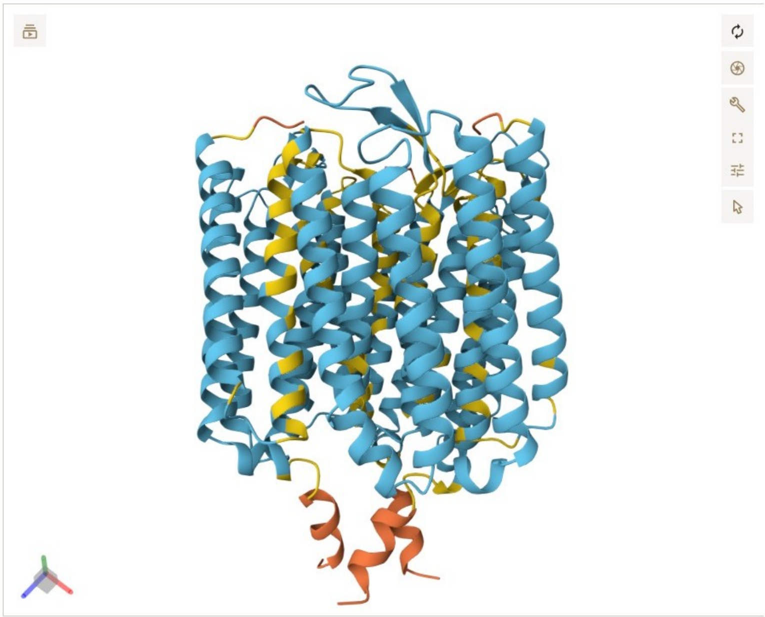

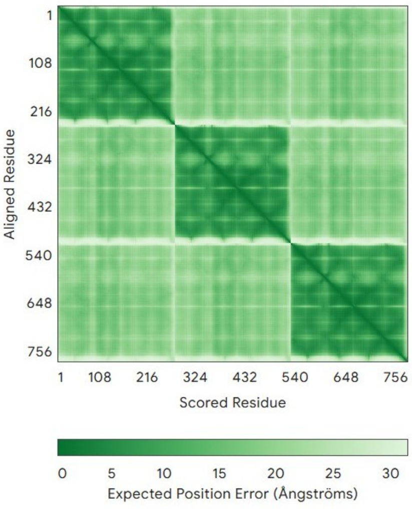

Non-commercial use only, subject to AlphaFold Server Output Terms of Use; no use in docking or screening tools.

Information

Type	Copies	Sequence
Protein	3	AVRENAQQSS SQWTNTAQAG TATQTYTYMG RTIRPGRPRQ TWGATQMTPQ TSTSSYQGQQ SGLTVGMIEM PAGHALAGEM VRSQWGRYQT WAQSTPMTQQ AQGQQADVDQ GSQYTTTAAD TGMCTTGQAA AMTTSAQQYR WAYYATSCAY YTTTQSAQTT DWAASASSAG TAETYDTQRT QTTTQWQGYP TTWATGVEGL ALVQSTGTTS WAYSTQDTYA KYTYAYTQQR WTANNERTVA VAGQTLGTMS SDD (10, 20, 30, 40, 50, 60, 70, 80, 90, 100, 110, 120, 130, 140, 150, 160, 170, 180, 190, 200, 210, 220, 230, 240, 250, 253)

Seed: 2140670312

o) ChR2^{QTY} dimer

Q8RUT8_QTY_dimer

← Back   Download   Clone and reuse   Feedback on structure

Very high (pIDDT > 90)   Confident (90 > pIDDT > 70)   Low (70 > pIDDT > 50)   Very low (pIDDT < 50)

ipTM = 0.79   pTM = 0.8   learn more

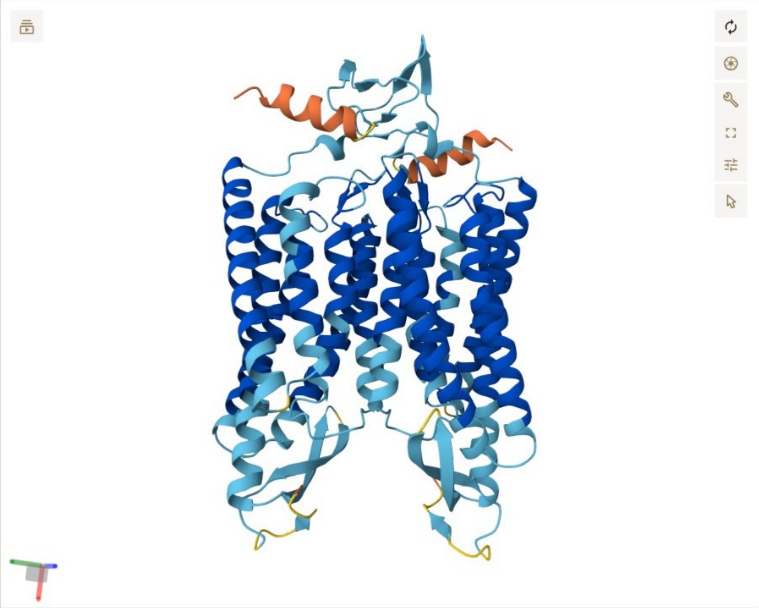

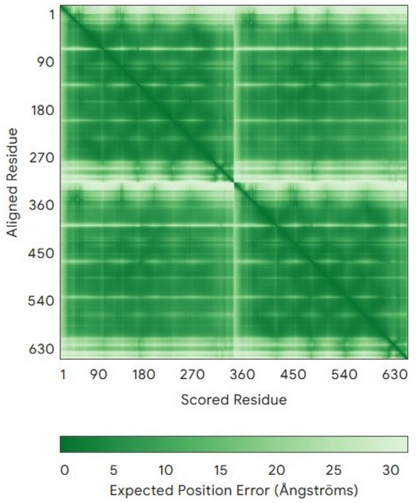

Non-commercial use only, subject to AlphaFold Server Output Terms of Use; no use in docking or screening tools.

Information

Type	Copies	Sequence
Protein	2	MDYGGALSAV (10) GRELLFVTNP (20) VVVNGSVLVP (30) EDQCYCAGWI (40) ESRGTNGAQT (50) ASNTQQWQAA (60) GYSTQQQMYY (70) AYQTWKSTCG (80) WEETYTCATE (90) MTKTTQEYYY (100) EFKNPSMLYL (110) ATGHRVQWQR (120) YAEWQQTCPT (130) TQTHQSNLTG (140) LSNDYSRRTM (150) GQQTSDTGTT (160) TWGATSAMAT (170) GYTKTTYYCQ (180) GQCYGANTYY (190) HAAKAYTEGY (200) HTTPKGRCRQ (210) TTTGMAWQYY (220) TSWGMYPTQY (230) TQGPEGFGVL (240) STYGSTTGHT (250) TTDQMSKNCW (260) GQQGHYQRTQ (270) THEHTQTHGD (280) IRKTTKLNIG (290) GTEIEVETLV (300) EDEAEAGAVN (310) KGTGK (315)

Seed: 1502359604

Figure S3. The radius of gyration of native and QTY-designed OPN2. By convention, the isomerization is set at time 0ns, which is indicated by a brown, vertical dashed line. The radius of gyration and its 1-ns running average are shown, respectively, as black and red lines.

a) Native OPN2

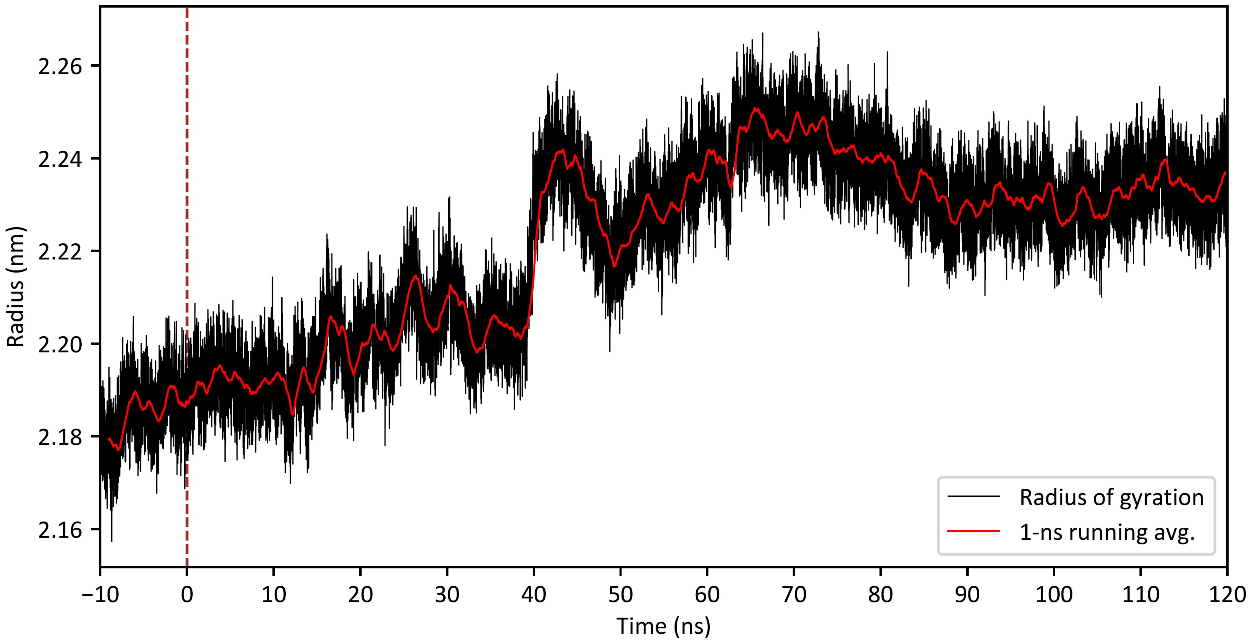

b) QTY-designed OPN2

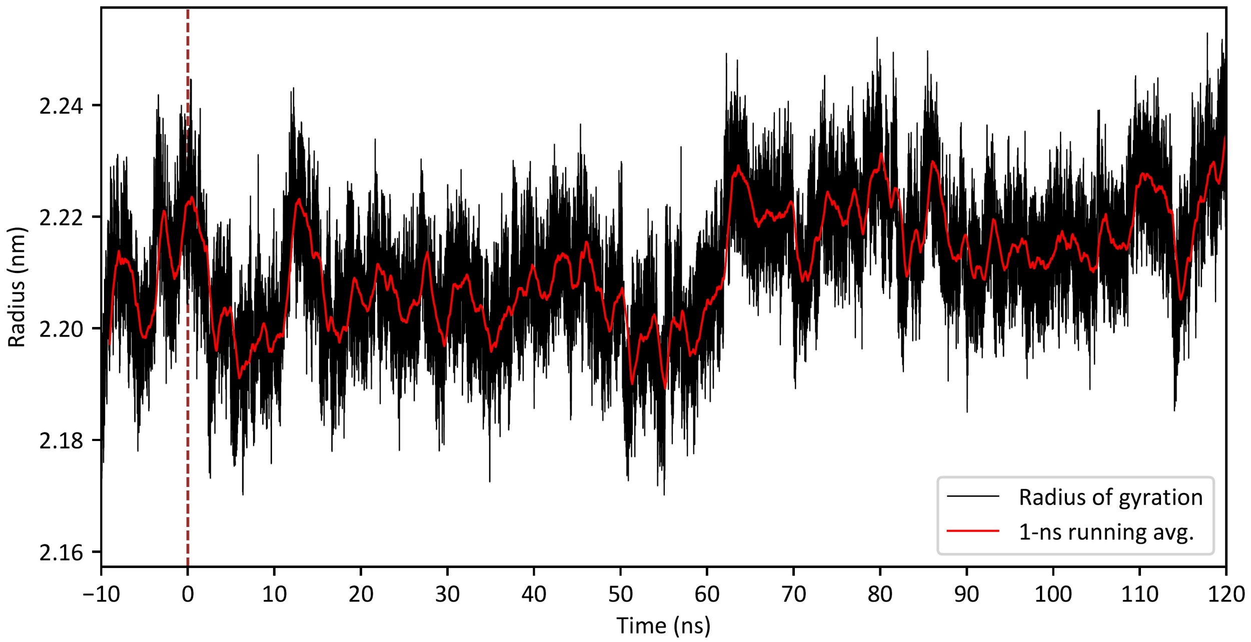

Figure S4. The root mean square (RMS) fluctuation of native and QTY-designed OPN2. Blue bars represent proteins with 11-cis-retinal and yellow bars represent proteins with all-trans-retinal. The two states are plotted on the same set of axis, with the shorter bars placed on top of the taller bars.

a) Native OPN2

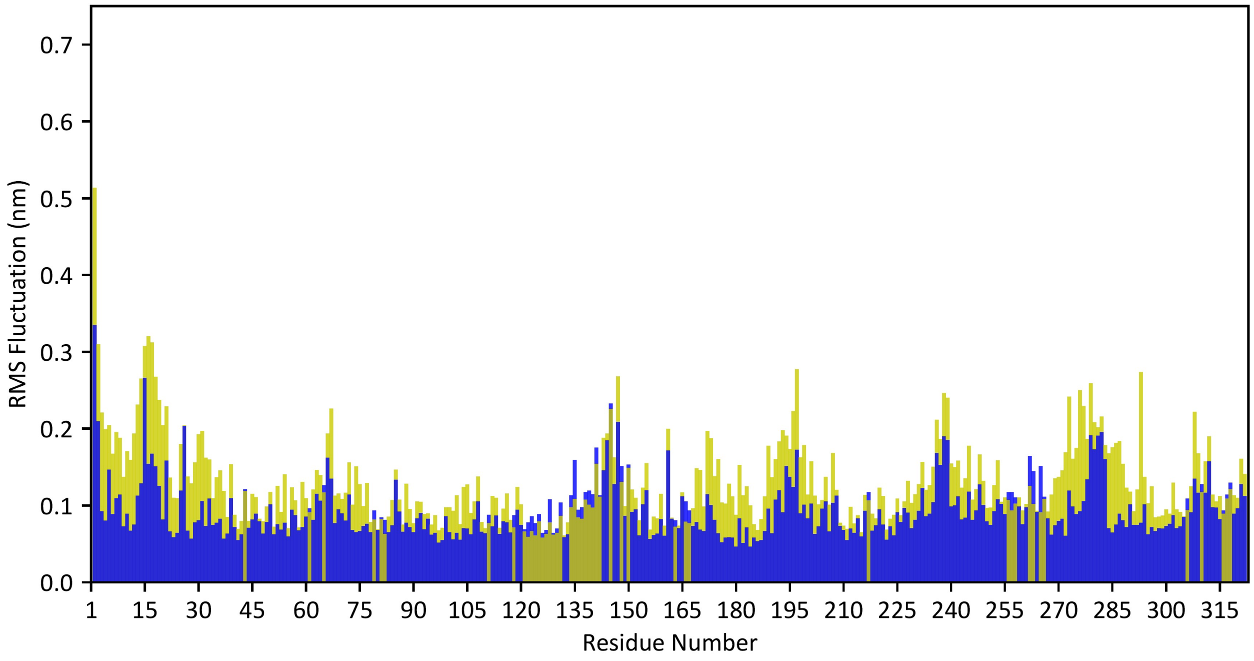

b) QTY-designed OPN2

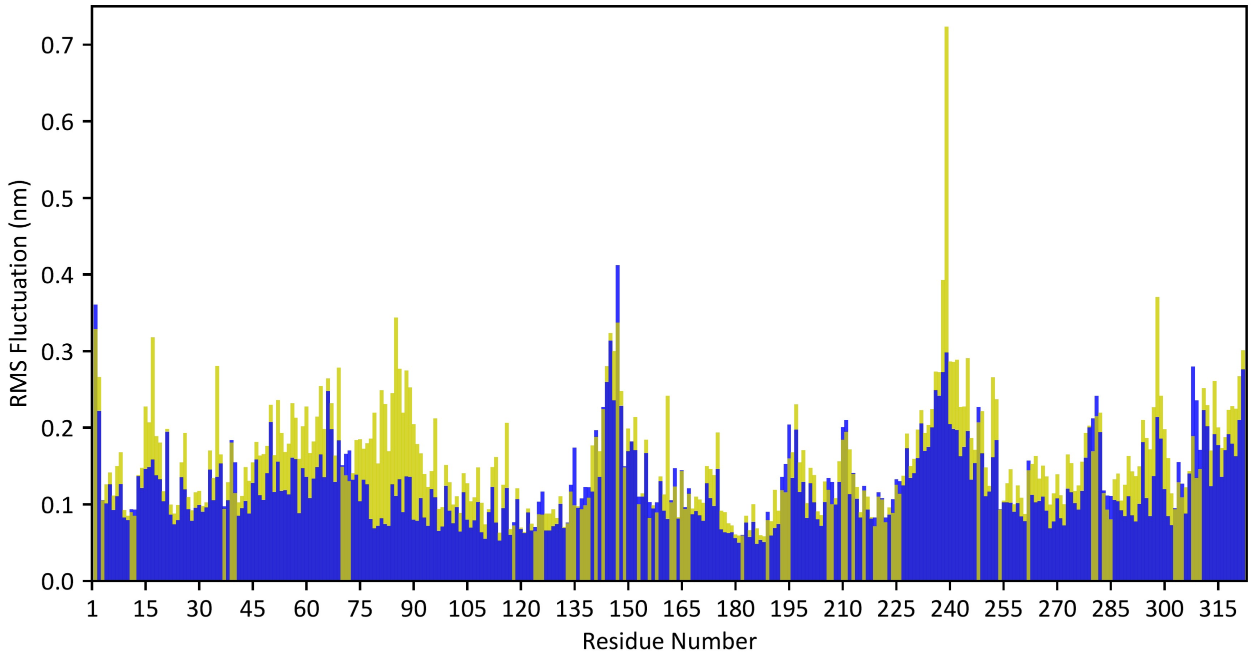

Figure S5. The root mean square distance (RMSD) of each residue in the retinal-binding pocket of native and QTY-designed OPN2. By convention, the isomerization is set at time 0ns, which is indicated by a brown, vertical dashed line. Only the 1-ns running average of RMSD are shown for clarity.

a) Native OPN2

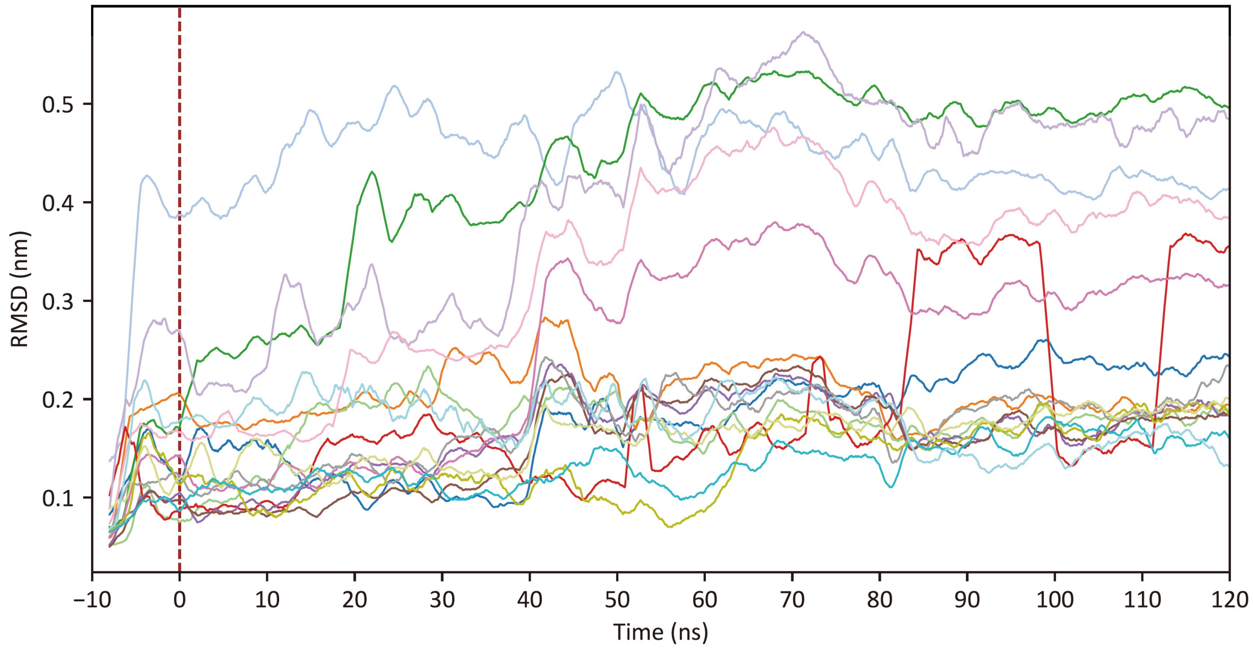

b) QTY-designed OPN2

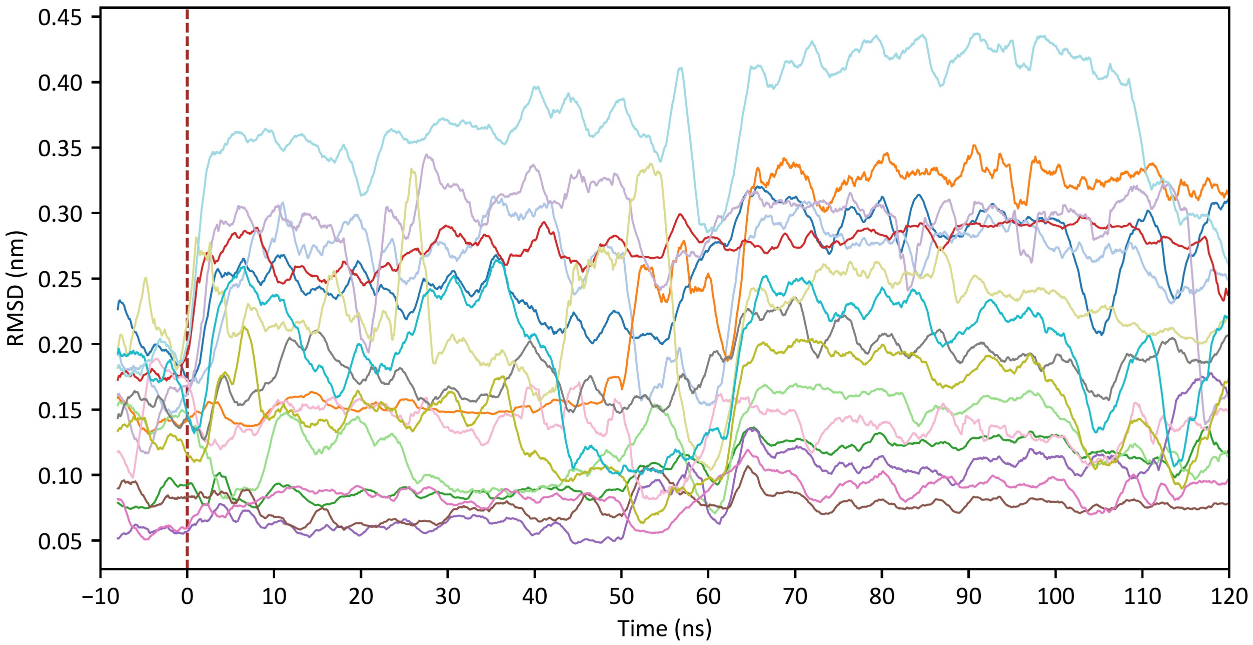

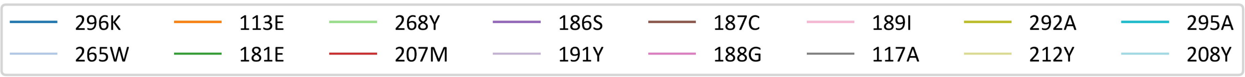

Supplement: Pan supplementary material [file S2633289225100094sup001.pdf]
