# Supplementary material for: A structural and functional bioinformatics study of QTY-designed retinylidene proteins
Source: QRB Discov. 2025 Jul 14;6:e20. doi: 10.1017/qrd.2025.10009 (PMC12361693; doi:10.1017/qrd.2025.10009)
Supplement: Peer review history

# Reviewer report: Review: A structural and functional bioinformatics study of QTY-designed retinylidene proteins — R0/PR2

**DOI:** 10.1017/qrd.2025.10009.pr2

This manuscript presents a bioinformatic study of the structure and properties of retinylidene proteins and their QTY-designed variants using AlphaFold3. Both the natural OPN2 and its QTY-designed variant were analyzed using AlphaFold3 and molecular dynamics simulations to investigate their responses to the isomerization of 11-cis-retinal to all-trans-retinal. The study is well-conducted and suitable for publication in QRB Discovery following revision.

1. The manuscript could provide more background and discussion on the downstream signaling pathways of OPN2. What is the potential significance for the treatment of related diseases and the underlying mechanisms of signal transduction？

2. The current MD analysis mainly focuses on structural changes, but it is recommended to include more targeted analysis of functionally relevant regions. Additionally, please clarify how the molecular dynamics simulation setup relates to real physiological processes.

3. In Figure 5, the RMSD of the protein and retinal in the QTY analog appears to be still fluctuating. If longer simulations are not feasible, it is recommended to include statistical analyses over different time windows to demonstrate whether the structure is being stabilized.

4. Please clarify the role and significance of the reverse-QTY design mentioned at the end of the introduction in the context of this study.

5. The first citation of Karagol, A. on page 8 contains garbled characters. This journal name of this citation is also not consistent with others. The citation in the third line of page 9 is incomplete. Please check all reference formats throughout the manuscript.

6. The uses of past and present tenses are not consistent. The manuscript would benefit from editing by a native speaker.

# Reviewer report: Review: A structural and functional bioinformatics study of QTY-designed retinylidene proteins — R0/PR3

**DOI:** 10.1017/qrd.2025.10009.pr3

This study applies a water solubilization design method called QTY code to monoamine transporters, computationally characterizing some properties of the designed proteins and investigating the relationship between natural mutations in these monoamine transporters and the QTY code. These findings are intriguing and hold some significance for the study on monoamine transporters and the QTY code; however, there are some issues in the manuscript should be addressed by the authors.

# Editor report: Recommendation: A structural and functional bioinformatics study of QTY-designed retinylidene proteins — R0/PR4

**DOI:** 10.1017/qrd.2025.10009.pr4

**Affiliations:** Chemistry, Chalmers University of Technology, Sweden

No accompanying comment.

# Editor report: Decision: A structural and functional bioinformatics study of QTY-designed retinylidene proteins — R0/PR5

**DOI:** 10.1017/qrd.2025.10009.pr5

**Affiliations:** Chemistry, Chalmers University of Technology, Sweden

No accompanying comment.

# Reviewer report: Review: A structural and functional bioinformatics study of QTY-designed retinylidene proteins — R1/PR7

**DOI:** 10.1017/qrd.2025.10009.pr7

The author resolved my questions and I think the manuscript is good for acceptance.

# Editor report: Recommendation: A structural and functional bioinformatics study of QTY-designed retinylidene proteins — R1/PR8

**DOI:** 10.1017/qrd.2025.10009.pr8

**Affiliations:** Chemistry, Chalmers University of Technology, Sweden

No accompanying comment.

# Editor report: Decision: A structural and functional bioinformatics study of QTY-designed retinylidene proteins — R1/PR9

**DOI:** 10.1017/qrd.2025.10009.pr9

**Affiliations:** Chemistry, Chalmers University of Technology, Sweden

No accompanying comment.
